# Fibrous Aerogels with Tunable Superwettability for High-Performance Solar-Driven Interfacial Evaporation

**DOI:** 10.1007/s40820-023-01034-4

**Published:** 2023-03-10

**Authors:** Chengjian Xu, Mengyue Gao, Xiaoxiao Yu, Junyan Zhang, Yanhua Cheng, Meifang Zhu

**Affiliations:** State Key Laboratory for Modification of Chemical Fibers and Polymer Materials, College of Materials Science and Engineering, Donghua University, Shanghai, 201620 People’s Republic of China

**Keywords:** Cellulose aerogel, Tunable wettability, Thermal insulation, Robust interface, Solar vapor generation

## Abstract

Solar-driven interfacial evaporation is an emerging technology for water desalination. Generally, double-layered structure with separate surface wettability properties is usually employed for evaporator construction. However, creating materials with tunable properties is a great challenge because the wettability of existing materials is usually monotonous. Herein, we report vinyltrimethoxysilane as a single molecular unit to hybrid with bacterial cellulose (BC) fibrous network, which can be built into robust aerogel with entirely distinct wettability through controlling assembly pathways. Siloxane groups or carbon atoms are exposed on the surface of BC nanofibers, resulting in either superhydrophilic or superhydrophobic aerogels. With this special property, single component-modified aerogels could be integrated into a double-layered evaporator for water desalination. Under 1 sun, our evaporator achieves high water evaporation rates of 1.91 and 4.20 kg m^−2^ h^−1^ under laboratory and outdoor solar conditions, respectively. Moreover, this aerogel evaporator shows unprecedented lightweight, structural robustness, long-term stability under extreme conditions, and excellent salt-resistance, highlighting the advantages in synthesis of aerogel materials from the single molecular unit.

**Supplementary Information:**

The online version contains supplementary material available at 10.1007/s40820-023-01034-4.

## Highlights

- Hybrid fibrous aerogels with tunable wettability from the same molecular unit of vinyltrimethoxysilane are successfully developed.
- Superhydrophobic and superhydrophilic hybrid aerogels are integrated into a double-layered evaporator, showing robust interfacial networks to withstand repeated and tremendous compression for 1000th cycle.
- The evaporator delivers high water evaporation rates of 1.91 kg m^−2^ h^−1^ under laboratory conditions and 4.20 kg m^−2^ h^−1^ under outdoor experiments with the aid of wind (1 sun), enabling efficient salt rejection under continuous operation.

**Supplementary Information:**

The online version contains supplementary material available at 10.1007/s40820-023-01034-4.

## Introduction

Freshwater scarcity is becoming a threat to the sustainable development of human society [1, 2]. Steam and clean water generation from either seawater or wastewater, assisted by the application of solar evaporation, is one of the most potential green and sustainable strategies to relieve global water shortages [3, 4]. Currently, the majority of efforts at this frontier are centered on interfacial heating-based evaporation, which is proposed to confine heat at the liquid surface and has successfully boosted evaporation efficiency [5–8]. Compared with traditional evaporation of bottom heating and bulk heating, interfacial evaporation selectively heats the evaporative part of water rather than the entire body of water [9–12].This technology is promising to expand the utilization of solar-thermal technologies in stand-alone, compact, and portable systems [13, 14]. In general, interfacial evaporation systems usually have double-layered structures with separate surface wettability properties on the top and bottom layers [15]. Hydrophilic top layer loading solar absorbers continuously pumps the underlying water for vapor evaporation, while the hydrophobic bottom layer enables the evaporator to float on the surface of water as well as to suppress the heat loss of converted energy to the bulk liquid and surrounding environment. To date, various materials have been explored, including cotton fabrics [16, 17], electrospun nanofibers [18, 19], graphene oxide membranes [20, 21], polymer foams [22–24], and aerogel materials [15, 25, 26]. Among them, aerogel materials show more advantages because of their lightweight, excellent thermal insulation, and interconnected porous water channel [27–30].

Cellulose nanofibers are excellent building blocks for aerogel construction, which are highly crystalline with a diameter of 4 to 100 nm and a length of roughly several micrometers, and featured an attractive combination of biocompatibility, high mechanical robustness, low density, and flexible surface chemistry [31–34]. Due to a high aspect ratio of cellulose nanofibers and their interpenetrate network, nanofibers could be assembled into ultralight and highly porous aerogels [35, 36]. Owing to the weak intra-and inter-molecular hydrogen bonds between pristine cellulose fibrous network, chemical crosslinking is extensively used to strengthen the network for robust aerogels [37]. Besides, these approaches could simultaneously endow the as-prepared aerogels with different wetting behavior, depending on the chemical structure of the corresponding binding agent. For example, epichlorohydrin and hexamethyldisilazane (or methyltrimethoxysilane (MTMS)) were reported as crosslinkers to respectively endow the cellulose aerogels with hydrophilicity and hydrophobicity, which were then assembled into a double-layered aerogel device for solar vapor generation [25, 26]. However, existing chemical modification for aerogel wettability tuning is often monotonous. Heterogeneous bridging of the aerogels with distinct surface wettability using incompatible molecular binders usually results in weak interfacial bonding and even delamination between two adjacent layers [38–40]. Therefore, constructing a hydrophilic-hydrophobic interface based on a single molecular unit would be highly desirable for structural integrity and long-term stability.

Nature has long been a source of inspiration for the creation of materials with tunable properties and functions based on supramolecular assembly [41]. For example, amphiphilic protein manupulates wettability through controlling the assembly pathways of hydrophilic and lipophilic segments. Hydrophilic globular proteins could be stabilized in water by burying hydrophobic side chains in the interior of a globular protein, and exposing hydrophilic segments on the surface of the globular proteins to contact with water. Reversely, membrane proteins having hydrophobic side chains oriented outward and hydrophilic segments buried inside show hydrophobic property [42]. We hypothesized that such assembly-driven strategy could also be applied to design aerogels with distinct hydrophobicity based on a single molecular unit, endowing double-layered aerogels with robust hetero-interface for high-performance water desalination.

Herein, we choose vinyltrimethoxysilane (VTMS) as a single molecular unit to strengthen bacterial cellulose (BC) fibrous network, constructing aerogels with tunable wettability mediated by assembly pathways. Siloxane groups or carbon atoms are exposed on the surface of BC nanofibers, resulting in either superhydrophilic or superhydrophobic aerogels. A pronounced difference in wettability of as-prepared aerogels allow them to assemble into a double-layered solar evaporator by addition of photothermal materials, which achieves the evaporation rate of 1.91 kg m^−2^ h^−1^ and efficiency of 85% under 1 sun. In this system, superhydrophobic aerogels allow the whole evaporator floating on the water, and could act as an insulator to minimize the loss of the converted solar heat to the bulk water; while superhydrophilic aerogels allow sufficient water supply for highly efficient interfacial evaporation, and facilitate ions diffusion for salt-resistance solar desalination. Moreover, owing to the utilization of the single molecular unit, the interfacial networks that bridge adjacent aerogels are mediated mainly by physical entanglement and chemical bonding between polysiloxane and BC nanofibers, which offering unprecedented combination of structural integrity, simple preparation, and low cost, and will provide new strategies for practical applications of water purification.

## Experimental Section

### Reagents and Materials

BC hydrogels were purchased from Hainan Yide Co. Ltd., China. Vinyltrimethoxysilane (VTMS) (98%) was bought from Shanghai Macklin Biochemical Co. Ltd., China. Di-tert-butyl peroxide (DTBP, 98%), pyrrole (99%), tert-butanol (TBA, 99%), acetic acid (~ 95%), and ammonium persulphate (APS, 98%) were bought from Shanghai energy chemical Co. Ltd., China. Hydrochloric acid (HCl) (36.0 ~ 38.0%) and hexadecylcetyltrimethylammonium bromide (CTAB, 99%) were bought from Sigma-Aldrich Co. Ltd., China. Sodium hydroxide (NaOH), sodium chloride (NaCl), calcium chloride (CaCl_2_), magnesium chloride (MgCl_2_), magnesium sulphate (MgSO_4_), potassium chloride (KCl), sodium bromide (NaBr), and sodium bicarbonate (NaHCO_3_) were bought from Sinopharm chemical reagent Co. Ltd., China. The aggregation induced emission (AIE) molecules were obtained from AIEgen Biotech Co. Ltd., China. All chemicals were of analytical grade and directly used without further purification.

### Preparation of Fibrous Aerogels

#### Aerogels Preparation

Free radical polymerization of VTMS was prepared in the presence of radical initiator of DTBP. The reaction temperature, polymerization time, and DTBP concentration were controlled to be 120 °C, 48 h, and 10 mol%, respectively. The molecular weight of the resulted polymers (PVTMS) was tested by Gel Permeation Chromatography (GPC, Agilent PL-GPC220) and the mass average molar mass is 9.3 × 10^3^ g mol^−1^. Aerogels were fabricated by freezing-drying process as follows. Firstly, BC nanofibrous aqueous dispersion in a concentration of 0.3 wt% was prepared according to our previous work [43]. Meanwhile, 0.5 mL of VTMS was added into a mixed solution of water, TBA, and acetic acid (v/v/v: 10:10:3) under vigorous stirring for 30 min. Then, the above siloxane sol solution was mixed with BC nanofiber solution to obtain an intermediate homogenous mixture. Freeze-drying process was followed to develop superhydrophobic VTMS-based nanofibrous aerogels (VNFs). Superhydrophilic PNFs was developed through the same conditions with that of VNFs, but using PVTMS as precursor.

#### Preparation of AIE-Dopped Aerogels

To prepare AIE-dopped aerogels, hydrophobic TPE (or hydrophilic TPE-am) was homogenously mixed with VTMS (or PVTMS) in a molar ratio of 1:5000, yielding AIE-containing siloxane solution. Then, a similar aerogel preparation process was followed to obtain AIE-dopped aerogels. In this system, AIE molecules were confined within the polysiloxane network of VNFs and PNFs aerogels.

#### Preparation of Superposed Double-Layered Solar Evaporator

A superposed double-layered solar evaporator was developed by integration of VNFs and PNFs through sequential freeze process, in which both aerogels were vertically aligned to the evaporator through one-step freeze-drying process. In order to introduce photothermal materials into PNFs, PPy was *in-situ* grown within BC nanofibers network to prepare PNFs-PPy. Firstly, 0.50 mL of pyrrole monomer, 1.70 g of CTAB, and BC nanofibers (0.3 wt%) were dissolved in 125 mL of HCl (0.2 M). Then, the mixture was put at low temperature under vigorous stirring, followed by addition of the oxidizing agent of APS to initiate polymerization of pyrrole [44]. After repeated filtration and washing process, the BC-PPy mixture was obtained. Then, a similar PNFs aerogel preparation process was conducted to obtain PNFs-PPy aerogel.

### Characterization

The chemical structure of polysiloxane were studied by solid-state ^29^Si NMR spectra (Bruker AVANCE III 400 MHz spectrometer). Attenuated total reflection-Fourier transform infrared spectrometry (ATR-FTIR, Nicolet 6700) and X-ray photoelectron spectroscopy (XPS, Thermo ESCALAB 250) measurements of aerogels were carried out according to the procedures described previously [43, 45]. The internal and surface morphologies of VNFs and PNFs were studied by field-emission scanning electron microscopy (SEM, Hitachi SU8010). The nanostructure of hybrid fibrils within VNFs and PNFs were studied by high-resolution surface scanning transmission electron microscopy (STEM, Talos F200S) and energy-dispersive X-ray spectroscopy (EDS) mapping (Agar S106). Photoluminescence (PL) spectra were measured on a PTI QM/TM. Fluorescence excitation and emission wavelength were used as follows: TPE-am: *λ*_ex_ = 370 nm, *λ*_em_ = 474 nm; TPE: *λ*_ex_ = 350 nm, *λ*_em_ = 470 nm. The absorption of PNFs-PPy was measured by a UV–vis-NIR diffuse reflectance spectroscopy (UV3600). The water contact angle measurements were performed on an optical contact angle meter (Theta Flex, Biolin Instrument) at ambient temperature. The volume of water droplet was 3 μL. The dimension of the aerogel was 20 × 20 × 20 mm^3^. Specifically, three water droplets dyed with methylene blue were dipped on the surface of aerogels, and the corresponding photos were captured by a Canon digital camera. To obtain aerogels with different polycondensation degree, freeze-drying time was controlled. For mechanical tests, VNFs and PNFs with a size of 20 × 20 × 20 mm^3^ were used. The static compression tests, dynamic loading–unloading fatigue cyclic tests, and dynamic mechanical analysis were performed according to our previous work [43]. Compression tests of double-layered evaporators (height: 24 mm, diameter: 16 mm) were conducted using the same protocol.

### Thermal Conductivity Measurement

Thermal conductivities (*λ*, mW m^−1^ K^−1^) of VNFs and PNFs were measured by transient hot-wire (THW)-based thermal conductivity analyzer (XIATECH TC3000E). To study thermal conductivity of aerogels under high (95%) and low (30%) relative humidity (RH), VNFs and PNFs were placed in environmental chamber (WGDW-100L) for 48 h before measurement. To simulate the actual application scenarios, VNFs and PNFs were immersed in water for 8 h before measurement.

### Solar Vapor Generation Measurement

Solar vapor generation experiments were performed using a standard solar simulator (Oriel Newport 69911). Solar density was monitored using a light power meter (SM206-solar, Xinbao). Infrared imaging (Fotric 225) was used to record the temperature variation of the samples. High accuracy balance (FA 2004, 0.1 mg in accuracy) was used to record the mass change of the system during evaporation. Evaporation rates and efficiencies of the evaporator were calculated referring to the mass change. During which, the values of evaporation rates in the dark field were subtracted for efficiencies calculation. Room temperature and humidity were controlled to be 25 °C and 50%, respectively. Artificial seawater was prepared according to literature [46, 47]. Specifically, 13.4 g of NaCl, 0.577 g of CaCl_2_, 1.13 g of MgCl_2_, 1.62 g of MgSO_4_, 0.365 g of KCl, 0.140 g of NaBr, and 0.100 g of NaHCO_3_ were respectively weighted and then dissolved into 500 ml of deionized water before use. To explore ions concentration of artificial seawater before and after evaporation, the samples were collected for further inductively coupled plasma optical emission spectrometer (ICP-OES, Prodigy-ICP) analysis. The outdoor experiments were conducted from 10:00 to 18:00 on Aug. 07, 2022 in Songjiang, Shanghai.

## Results and Discussion

### Hybrid Aerogels with Tunable Wettability

**Fig. 1:**
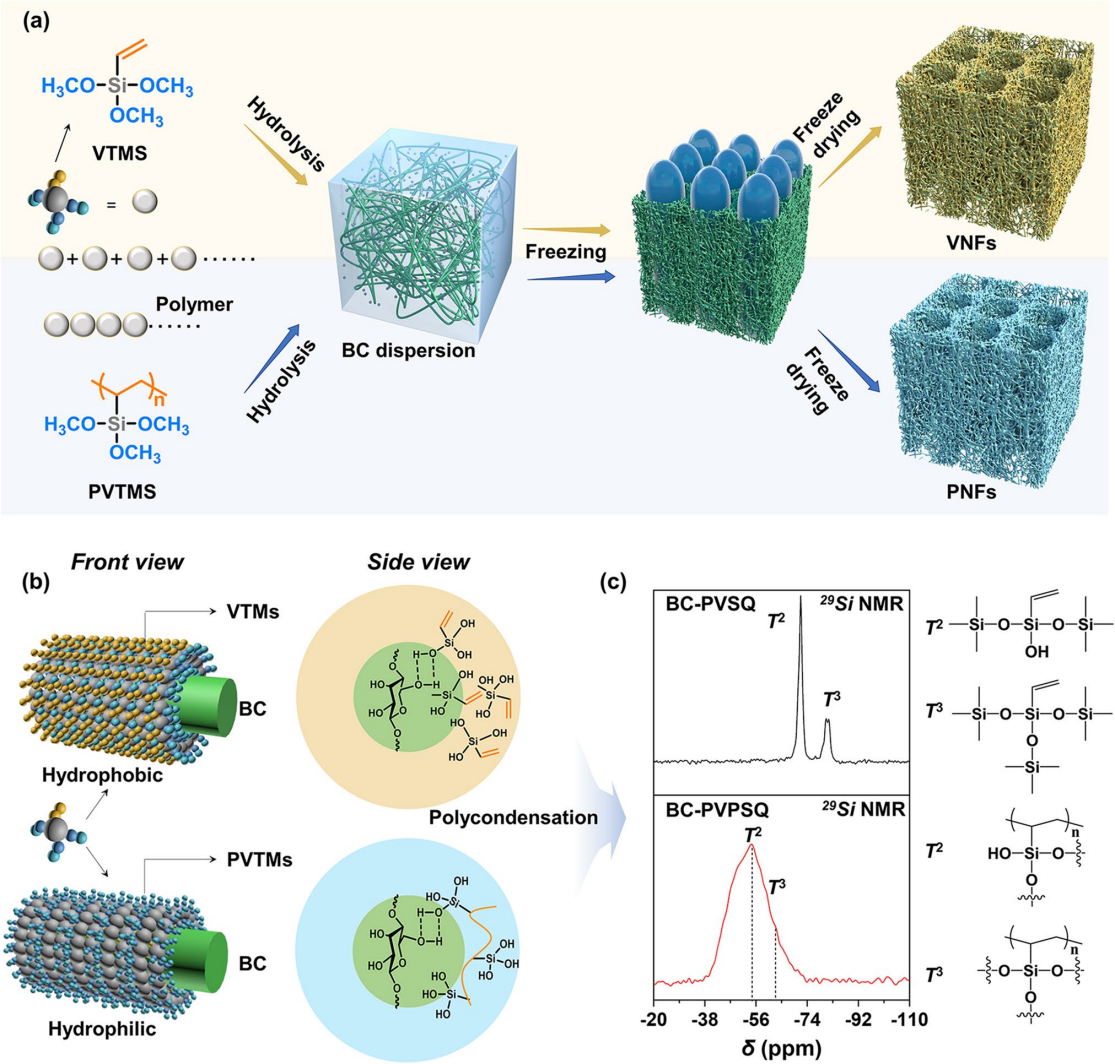
Processing principles and synthesis of the hybrid aerogels with tunable wettability. **a** Roadmaps of preparation of superhydrophobic VNFs and superhydrophilic PNFs. VTMS can be directly used or polymerized as precursors. Then the monomer (VTMS) or polymer (PVTMS) precursors are respectively mixed with BC nanofibers in desired mass ratios. After freezing and freeze-drying processes, polyvinylsilsesquioxane (PVSQ) and polyvinylpolysilsesquioxane (PVPSQ) are yielded to coat on the framework of BC nanofibers, resulting in VNFs and PNFs, respectively. **b** Molecular orientations of siloxane on the surface of BC nanofibers. **c** ^29^Si NMR spectra of PVSQ and PVPSQ, demonstrating the polycondensation of VTMS and PVTMS that promoted by freeze-drying process

The synthesis and fabrication roadmaps toward VNFs and PNFs are illustrated in Fig. 1a (also in Fig. S1) [43]. VTMS contains one polymerizable vinyl group (-CH = CH_2_) and three hydrolyzable methoxy groups (-OCH_3_). Firstly, materials of VTMS (or polymerized VTMS, namely PVTMS, Fig. S2) and BC nanofibers were mixed to obtain a homogenous dispersion. Next, the mixtures were added into acetic acid solution for hydrolysis process. Due to the hydrogen bonding between hydrophilic units of siloxane (Si–OH) and the hydroxyl groups (-OH) on the skeleton of BC nanofibers, hydrolyzed VTMS and PVTMS showed different pathways when assembling with BC nanofibers (Fig. 1b). Followed by freezing and freeze-drying process, polycondensation was occurred to yield polyvinylsilsesquioxane (PVSQ) and polyvinylpolysilsesquioxane (PVPSQ) on the surface of BC nanofibrous network, respectively, resulting in superhydrophobic VNFs and superhydrophilic PNFs. On the molecular level, more carbon atoms (-CH = CH_2_) are exposed on the surface of the BC nanofibers within VNFs whereas siloxane groups (Si–O–Si) are dominated the BC surface of PNFs. Both aerogels in cube form with an ultralow density of 6 ~ 8 mg cm^−3^ and a high porosity of ~ 95% show the ultra-lightweight character (Note S1, Fig. S3). The polycondensation process was monitored by ^29^Si NMR spectra (Figs. S4 and S5), where the formation of PVSQ and PVPSQ on BC nanofibers are demonstrated (Fig. 1c). Two main signals at − 72.0 and − 81.4 ppm assigned to two-(T^2^) and three-bridged (T^3^) siloxane network were observed from ^29^Si chemical shifts for PVSQ, while − 54.4 (T^2^) and − 63.0 (T^3^) ppm for PVPSQ [48, 49].

**Fig. 2:**
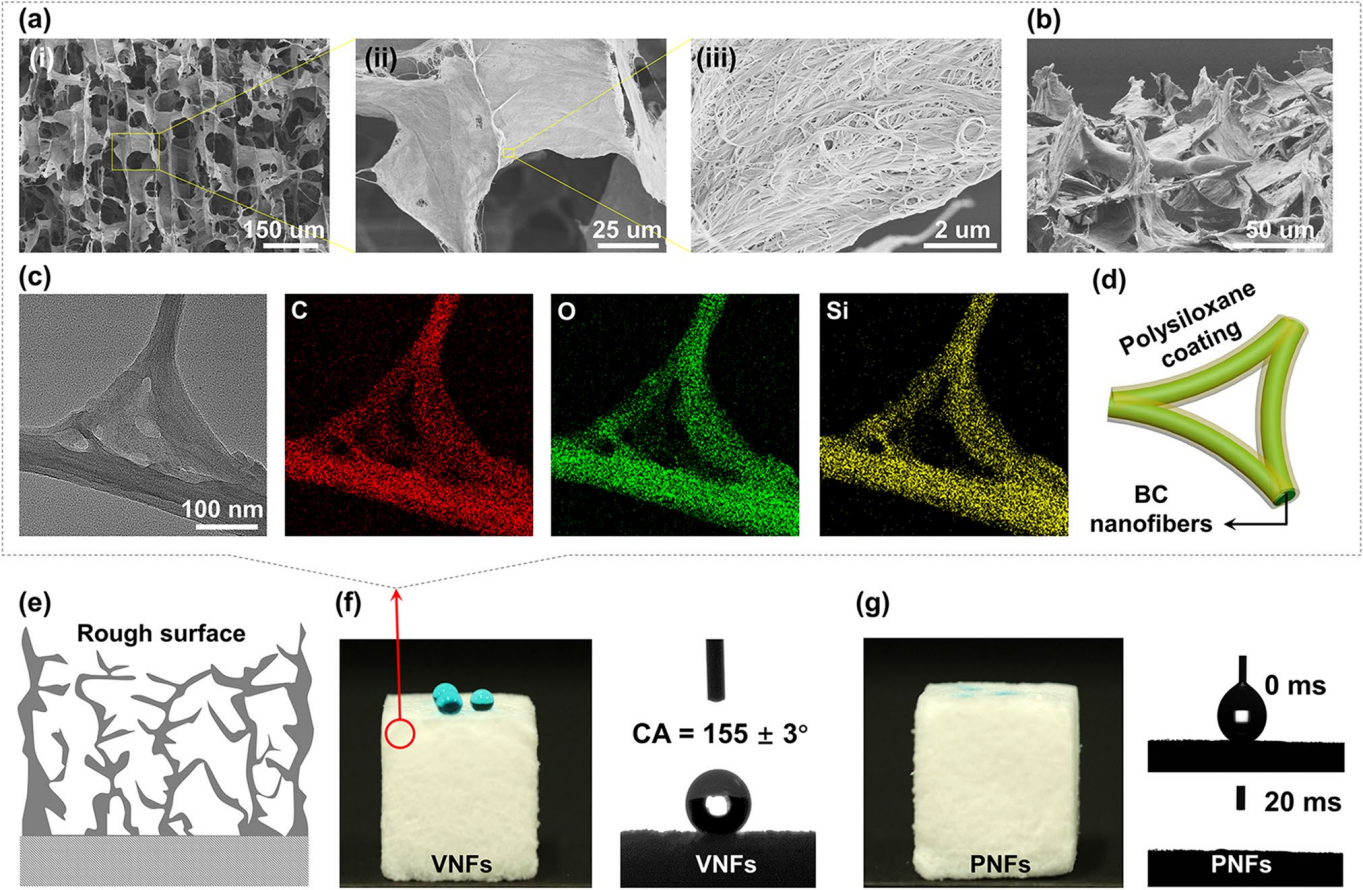
Microstructure and surface wettability of hybrid aerogels. **a** Microscopic structure of VNFs observed by SEM at different magnifications. **b** Side-view SEM image of superhydrophobic VNFs nanostructure. **c** STEM-EDS mapping of BC-PVSQ nanofibers with elements of C, O, and Si, respectively. **d** Schematic illustration of polysiloxane-coated BC nanofibers. **e** Surface roughness of the aerogels (VNFs and PNFs) with numerous nanoscale fibrous protrusions. **f** Digital photo of water drops on the surface of VNFs and corresponding contact angle measurements. **g** Digital photo of water absorbed on the surface of PNFs and corresponding contact angle measurements

SEM images show the interconnected and aligned cellular 3D fibrous framework within both aerogels (Figs. 2a–b and S6), while tightly entangled fibrous network could also be identified on fibrous cell walls. The direction of alignment is preferentially parallel to the movement of the freezing front of ice crystals [50]. From the study of STEM and energy-dispersive X-ray spectroscopy (EDS) mapping, polysiloxane of PVSQ and PVPSQ was demonstrated to homogeneously coat on the surface of BC nanofibers (Figs. 2c–d and S6) [43, 45]. The polysiloxane coatings not only strengthened the porous structures but also modified the wettability as compared with neat BC aerogels. The apparent water contact angle of 63° and 104° was observed for individual-component of PVSQ and PVPSQ (Fig. S7), respectively. The results indicate the distinct wettability between PVSQ and PVPSQ, where the intrinsic wetting threshold of water is 65° [51]. Not only the chemical compositions of materials, the surface microstructure is also accounted for the superwettability of the as-prepared aerogels. On the surface of the aerogels, the observed highly porous nanostructures and randomly distributed fibrous protrusions (Fig. 2b) can enhance both hydrophobicity and hydrophilicity. The rough surface (Fig. 2e) makes originally hydrophobic surface more hydrophobic (Fig. 2f, 155 ± 3° contact angle of water) and more hydrophilic (Fig. 2g, complete spreading of water in 20 ms) if the surface is intrinsically hydrophilic [52, 53].

### Mechanism Analysis and Visualization of Tunable Wettability

**Fig. 3:**
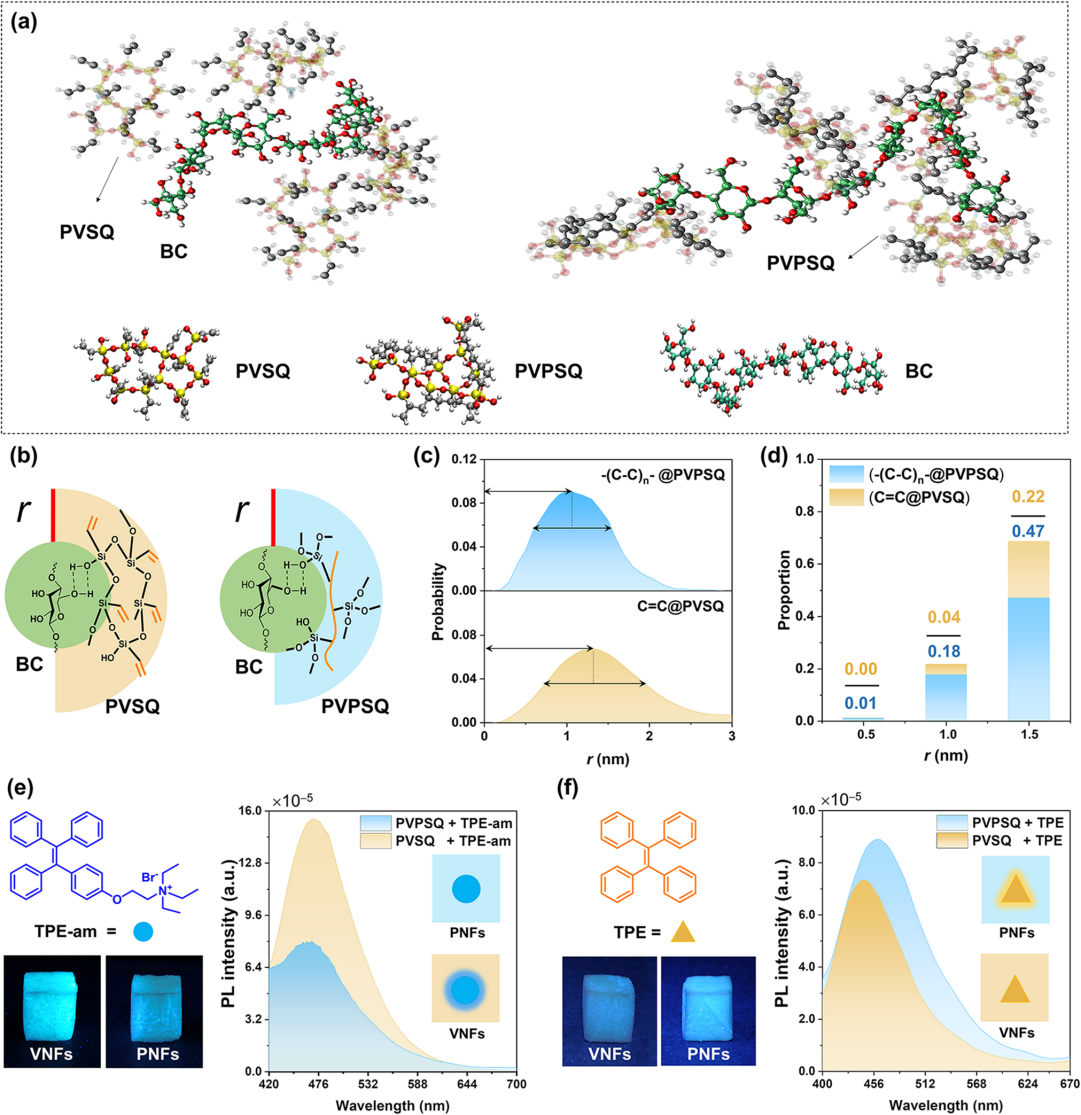
Mechanism analysis and visualization of tunable wettability. **a** A snapshot of molecular dynamics simulation of interactions between PVSQ/PVPSQ and BC nanofibers. **b** Molecular orientations of polysiloxane on the surface of BC nanofibers. The radial distance between the carbon atoms of PVSQ/PVPSQ and BC nanofibers is denoted as *r*.** c** The distribution of radial distances (*r*) between the carbon atoms of PVSQ/PVPSQ and BC nanofibers. **d** Proportion of carbon atoms for PVSQ and PVPSQ when *r* is 0.5 nm, 1.0 nm, and 1.5 nm, respectively. **e−f** AIE-doped aerogels to visualize the internal and integral wettability of the aerogels. Fluorescence images and spectra were taken under UV irradiation

To provide insight into the self-assembled structure of VNFs and PNFs, the orientation of PVSQ/PVPSQ molecules on BC nanofibers at the molecular scale were studied by molecular dynamics simulation. The calculation was started by modeling the segments of PVSQ/PVPSQ and cellulose, which were respectively packed in the cubic simulation using the PACKMOL [54]. The analysis procedure and the related parameter setting are depicted in Note S2, and detailed parameter values were listed in Table S1. Note that we assumed the cutoff radius (1.12 nm) between the BC nanofibers and polysiloxane (PVSQ or PVPSQ) as a criterion to distinguish the surface chemistry and the related wettability behavior of VNFs and PNFs [55]. After energy minimization and optimization process, the radial distances (*r*) between polysiloxane molecules and BC nanofibers are shown in the snapshots (Fig. 3a). The closer proximity between carbon atoms of PVPSQ molecules and BC nanofibers is observed than those of PVSQ molecules and BC nanofibers (Fig. 3b). Furthermore, the distribution of radial distances (*r*) of two self-assembled structures within the models were schemed in Fig. 3c–d. The probability maximum of radial distance between the carbon atoms of PVSQ/PVPSQ and BC nanofibers is 1.32 and 1.00 nm, respectively. The results confirm the molecular orientation of polysiloxane that wrapped around BC nanofibers as depicted in Fig. 3b. Moreover, Fig. 3d shows the carbon atomic proportion when the distance between the carbon atoms of PVSQ/PVPSQ and surrounding bacterial cellulose is less than 0.5, 1.0, and 1.5 nm, demonstrating that vinyl groups of PVSQ are mostly oriented away from the BC nanofibers and exposed outside to show hydrophobic surface; whereas the alkyl chains of PVPSQ are buried inside the polysiloxane network to afford its apparent hydrophilic character. These results are in accordance with wettability of VNFs and PNFs aerogels on the basis of water contact angle measurements that shown in Fig. 2f–g.

In addition to the surface wettability, the interior and integral wettability behaviors of VNFs and PNFs were studied by using AIE technology. The isolated AIE molecules are weakly or non-emissive, but lighting-up when they aggregated. According to the working mechanism of restriction of intramolecular motions, the AIE technology provides a sensitive, accurate, and easy-readable way for examining the microenvironment of systems [56–59]. As shown in Fig. 3e–f, PVSQ and PVPSQ coatings on BC nanofibers were labeled with tetraphenylethylene (TPE, hydrophobic) and quaternary ammonium-grafted TPE (TPE-am, hydrophilic). Hydrophilic TPE-am molecules showed stronger emission in VNFs than those in PNFs (Fig. 3e). But opposite phenomena were observed when doping hydrophobic TPE molecules in both aerogels (Fig. 3f). According to the theory of like dissolves like, the intramolecular motions of AIE molecules could be activated when they are dispersed in the compatible microenvironment. Molecular motions could efficiently dissipate the excited-state energy through nonradiative decay channels, and then weakened the fluorescence of AIE molecules. Based on the above working mechanism, the increased fluorescence of AIE molecules in incompatible network could also be well explained. The AIE fluorescence is dependent on localized microenvironment due to the working mechanism of molecular motion, providing a direct method to visualize the interior wettability of VNFs and PNFs. The homogeneous fluorescence signals of the AIE-dopped aerogels indicate the uniform wetting behavior of the whole aerogels.

### Superposed Double-Layered Solar Evaporator Construction

The excellent mechanical performance of BC hybrid aerogels could be expected after crosslinking with polysiloxane. Both VNFs and PNFs aerogels could quickly recover to the original shape after compression, which is demonstrated by the stress–strain (*σ–ε*) curves (Fig. S8). Moreover, the hybrid aerogels also showed an excellent fatigue resistance after 1,000 cyclic compressions at the strain of 60% (Fig. S9). In addition to the excellent mechanical robustness and fatigue resistance, our aerogels also show durable mechanical performance to a wide temperature range of − 150 to 200 °C, holding great potential for application in stringent environments (Figs. S10 and S11). With high porosity, lightweight (density: ~ 6 mg cm^−3^), good compressibility, and excellent hydrophobicity, VNFs represents an attractive candidate of thermal insulation materials. Hydrophobic PVSQ coatings improve water resistance in thermal insulation, endowing highly porous VNFs with durable thermal insulation performance in aqueous environment, making them promising to act as a floating layer for a solar evaporator. Meanwhile, superhydrophilic PNFs with interconnected porous structure are fortunately suitable to load solar absorber, allowing sufficient water supply and efficient vapor transport through the front layer [60]. The combination of air-filled pores for thermal insulation and water-filled pores for water transportation is highly potential for evaporator systems.

**Fig. 4:**
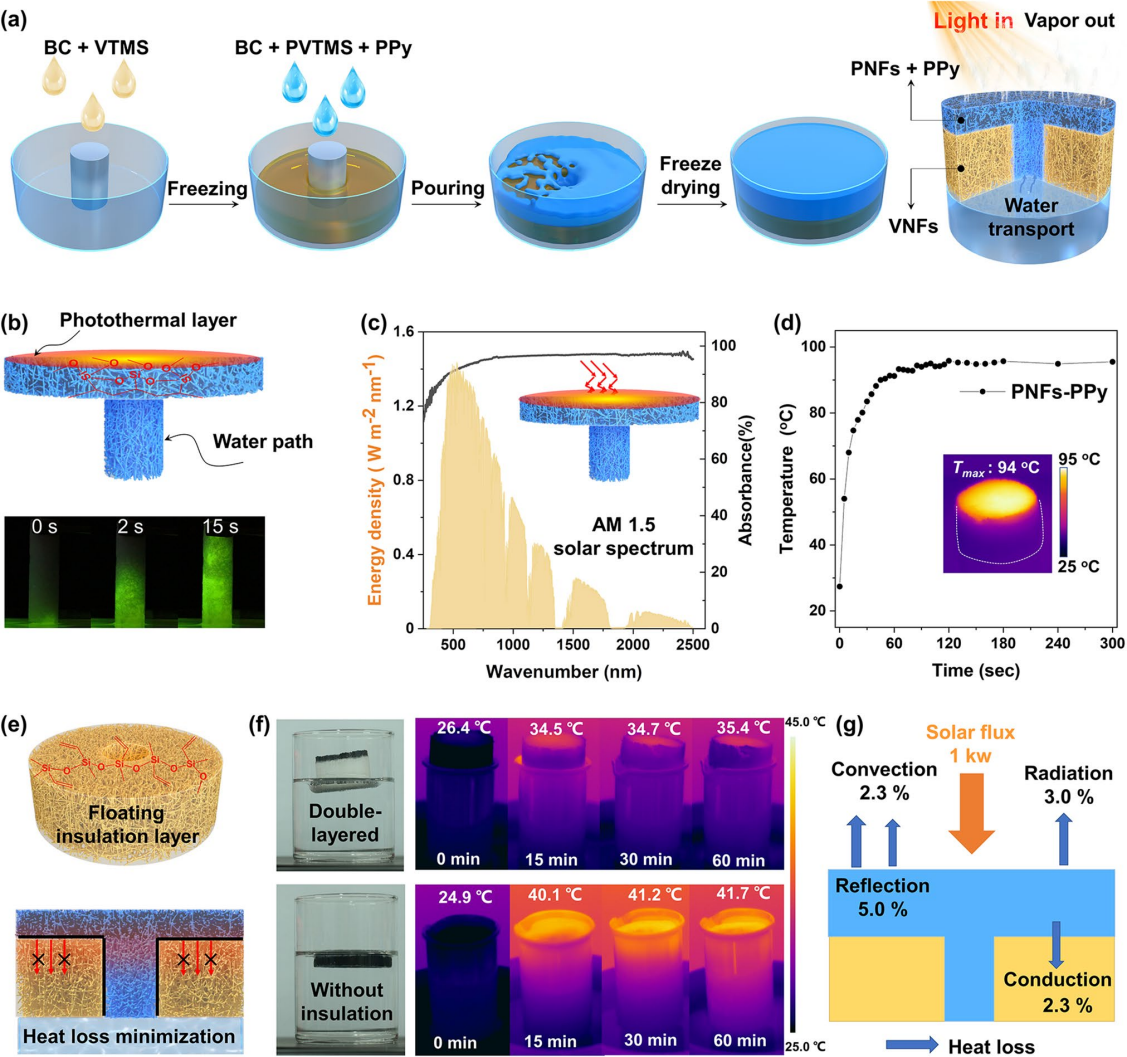
Superwettable aerogels for solar-driven interfacial evaporation. **a** A process flow scheme of evaporator device integration. **b** (Top) Schematic of the umbrella-shaped PNFs-PPy and (bottom) water diffusion of pure PNFs. **c** Absorption spectra of PPy-doped PNFs aerogel and solar irradiance weighted by standard AM 1.5G solar spectrum. **d** Temperature rise recorded from the top surface of PNFs-PPy upon 1 sun irradiation (1 kW m^−2^). **e** Schematic of a porous VNFs insulator that suppresses the downward heat loss. **f** Infrared images of double-layered evaporator and single-layered PNFs-PPy illuminated under 1 sun for 60 min. **g** A heat loss diagram of our evaporator

A superposed double-layered solar evaporator was developed by integration of VNFs and PNFs through sequential freeze process, in which both aerogels were vertically aligned to the evaporator through one-step freeze-drying process (Fig. 4a). In this case, the umbrella-shaped-PNFs loading with the solar absorbing materials (*e.g.*, polypyrrole (PPy) nanofibers) affords the structural elements of a solar absorber and a water pathway for the evaporation system. Figure 4b presents the rapid wetting process through pillared PNFs once in contact with water (labeled with fluorescein sodium). Based on capillary wicking effect, the vertical aligned porous structure and its superhydrophilic property boost the capillary pumping [25]. The PNFs showed negligible deformation in water based on the excellent mechanical elasticity, and the water transport distance of ~ 5.0 cm could be achieved in 15 s (Movie S1). The calculated water transport rate through PNFs-based water transportation channel could reach 2827 cm^3^ h^−1^ (Note S3), which is rather fast when compared with other porous materials [61–63].

Broadband light capture with efficient absorption and its high conversion into heat is another key factor to drive water evaporation [29]. PPy nanofibers are suitable for broadband solar absorption, and the synthesis and their doping in aerogels are illustrated in Fig. S12 [44]. As shown in Fig. 4c, PNFs doping with PPy nanofibers (PNFs-PPy) exhibited 95% absorption of solar irradiation in the range of 250–2500 nm, showing a strong and broad light absorption for efficient solar energy harvesting. Under 1 sun (1 kW m^−2^) irradiation, the surface temperature of the dry PNFs-PPy quickly rose from 27 °C to a steady state of 94 °C within 80 s (Fig. 4d). The inset shows the recorded infrared image of PNFs-PPy, disclosing the potential of PNFs-PPy for efficient heat conversion.

Confining solar-thermal heat at the air–water evaporative interface requires thermal insulation design, reducing heat conduction downward to bulk liquid and then improving evaporation performance [9]. Superhydrophobic VNFs show a stable thermal conductivity of 29.5 mW m^−1^ K^−1^ at high humid environment (relative humidity = 95%) and even after immersion in water for 8 h (Fig. S13). The low thermal conductivity of VNFs is attributed to their cellular micropores, nanopores, nanosized fibrous building blocks, and superhydrophobic character [64]. VNFs with a drilled hole could be integrated with umbrella-shaped PNFs to form a double-layered evaporator (Fig. 4e). In our double-layered floating evaporation system, porous VNFs could efficiently reduce the thermal leakage, restrict the generated heat diffusing to the bulk water or surrounding environment (Fig. 4f, top). In contrast, without a superhydrophobic VNFs bottom layer, the top solar-thermal converting layer of PNFs-PPy was fully wetted, accelerating heat conduction to the underlying water and ambient, strongly lowering the energy-conversion efficiency (Fig. 4f, bottom). As depicted in Fig. 4g, the double-layered evaporator with optimized geometry (will discuss below) presented a radiation loss of ~ 3.0%, a conduction loss of ~ 2.3%, and a convection loss of ~ 2.3% (Note S4) [15, 65]. The results demonstrate that VNFs could efficiently reduce heat loss and then improve evaporation efficiencies.

**Fig. 5:**
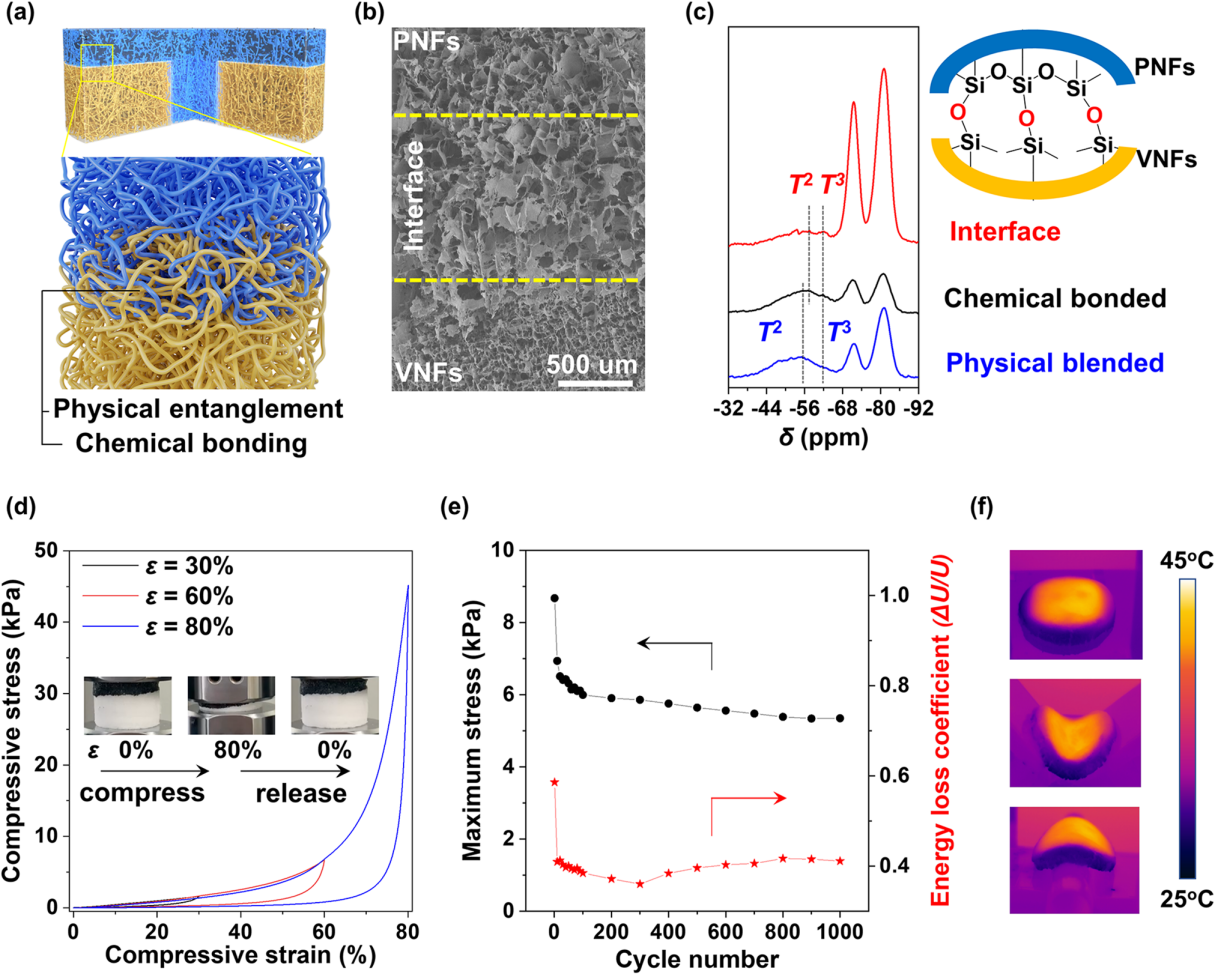
Robust interface for evaporator structural stability. **a** Schematic illustration of interface aera of the evaporator, showing physical entanglement between VNFs and PNFs. **b** SEM image of the evaporator interface area. **c** ^29^Si NMR spectra of polysiloxane at the interface area, chemically bonded PVSQ-PVPSQ, and physically blended PVSQ and PVPSQ. **d** Compressive stress–strain curves of the aerogel evaporator with incremental strains of 30%, 60%, and 80%, respectively. **e** Maximum stress and energy loss coefficient versus compressive cycles. **f** Infrared thermal images of our evaporator in either flat, inward bending, or outward bending

Implementation of evaporator system for practical applications also requires the long-term stability. Benefitting from our single-molecule strengthened interfacial strategy, our integrated evaporator shows robust interfacial networks that bridge adjacent aerogels with distinct wettability. As shown in the illustration of Fig. 5a, the representative network entanglement is indicated in the interface area of VNFs and PNFs. The morphology of the interface area was investigated by SEM image (Fig. 5b), smaller pores could be observed in the individual section of VNFs and PNFs, while larger pores in the interface area, generating a continuous morphology to combine the whole aerogel evaporator. Moreover, due to the utilization of the single molecule-based siloxane, during the freeze drying-induced polycondensation process, hydroxyl groups of siloxanes (Si–OH) may self-react to form crosslinked networks in the interface. As shown in Fig. 5c, ^29^Si NMR chemical shifts of the interface area, chemical bonded sample (− 57.0 ppm, T^2^), and physical blended sample (− 54.4 ppm, T^2^) are exhibited. Interface area shows characteristic chemical shift of the chemical bonded structure rather than the physical blended one, which is originated from the formation of covalent bonds between PVSQ and PVPSQ during hydrolysis and polycondensation [49, 66, 67]. Both physical entanglement and chemically bonded network afford the aerogel evaporator with robust interface, endowing the final aerogel evaporator with excellent mechanical integrity. Upon compression, the local buckling of the aerogel evaporator could be triggered due to good elasticity of VNFs and PNFs, and then recovered when released (Fig. 5d, Movie S2). The maximum compression stress of the evaporator at the strain of 80% is ~ 45 kPa, which is similar to the mechanical property of PNFs as PNFs is spanned within the whole evaporator. Moreover, after the first 3 conditioning cycles, the maximum stress was almost retained, and constant energy loss coefficient was calculated over 1,000 compress-release cycles (Figs. 5e and S14). Even in either flat or bending state, our aerogel evaporator still showed excellent structural integrity. The infrared thermal images in Fig. 5f demonstrate the uniform temperature distribution on our evaporator under sunlight irradiation, given the heat-conversion capability of the PNFs-PPy. Rooting in structural robustness (Fig. S15, Movie S3), efficient solar absorption, sufficient water supply, and excellent thermal insulation, the high performance of our superposed double-layered solar evaporator could be expected.

### Solar Evaporation Performance

**Fig. 6:**
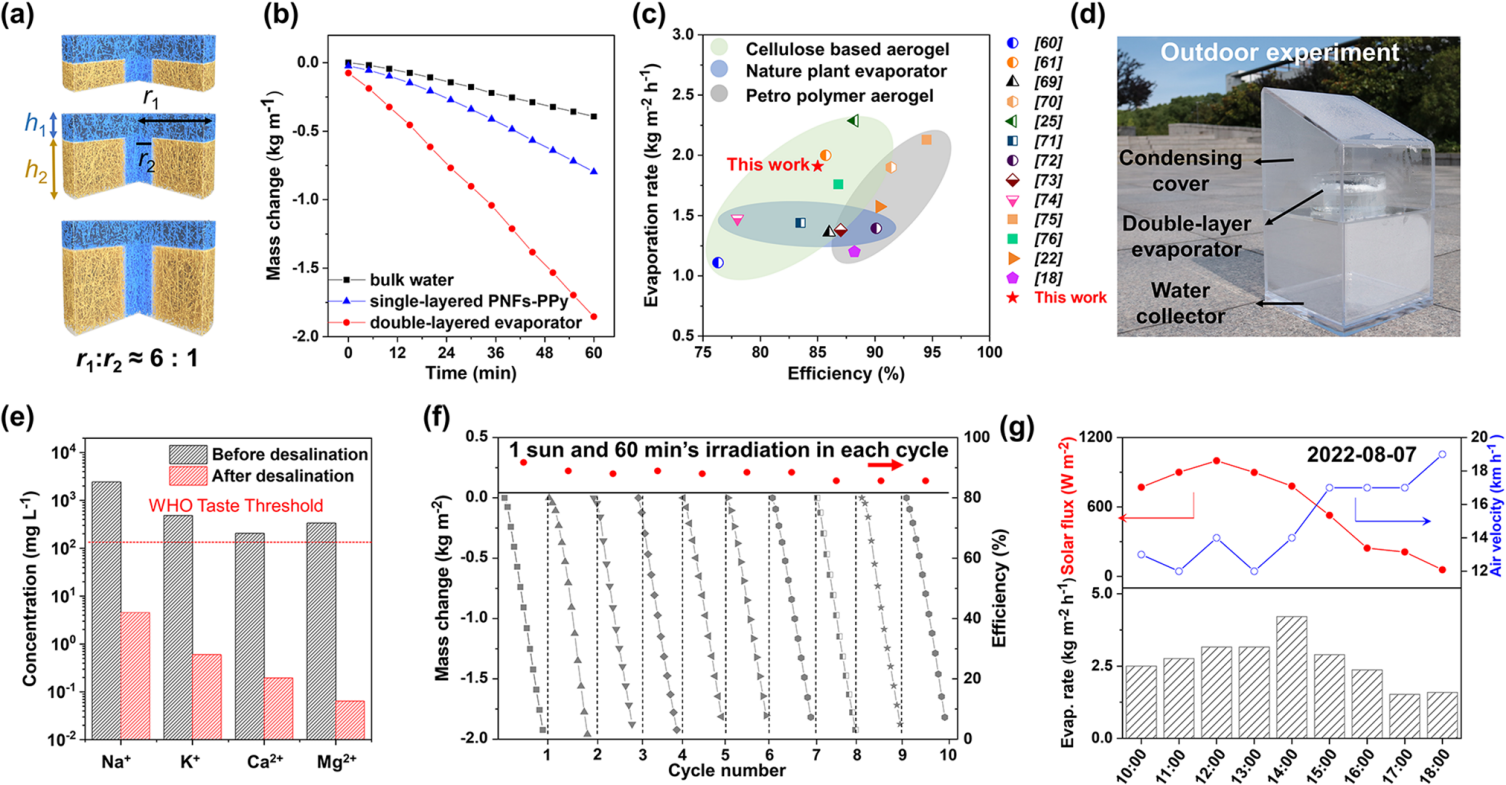
High-performance solar-powered interfacial evaporation. **a** Schematic drawings of superposed double-layered evaporators, where blue area represents PNFs-PPy and yellow area represents VNFs. **b** Time-dependent mass change of bulk seawater, single-layered PNFs-PPy, and double-layered evaporator under 1 sun irradiation. **c** Comparison of evaporation rates and efficiencies showing high performance of our evaporator. **d** Photograph of the setup for solar-driven water collection. **e** Four primary ion concentrations of a simulated seawater before and after desalination. **f** A long-term stability measurement of our evaporator over 10 h. **g** Water evaporation rates during 8 h of outdoor solar desalination with monitoring of sunlight intensity and air velocity

The geometry of the evaporator was optimized to achieve high-performance solar-to-vapor conversion. According to previous studies, the ratio of the diameters of top layer and pillar part was firstly determined as 6:1 (Fig. 6a), which endows the evaporator with thermal concentration capability for minimizing heat conduction loss to bulk water [27]. Next, the heights of top evaporative layer (*h*_1_) and bottom insulation part (*h*_2_) were optimized through evaluating the evaporation rates and evaporation efficiencies (Fig. S16). Evaporation efficiency (efficiency of solar to vapor generation) can be expressed as Eq. (1):

1 $$\eta = mh_{lv} /C_{opt} q_{solar}$$

where *m* is the evaporation rate, *h*_lv_ is calculated from equivalent evaporation enthalpy measurement (Note S5), *C*_opt_ the optical concentration and *q*_solar_ is the incident solar intensity (1 kW m^−2^). After recording the weight change of the evaporators under solar illumination, the optimized *h*_1_ of 1.0 cm and *h*_2_ of 2.0 cm were accurately determined, respectively. The optimized geometry is also supported by the calculation of the gravity, heat location and salt rejection performance of the structural elements (Note S6) [15, 68]. Figure 6b shows the typical plots of time-dependent mass change of the evaporators. The evaporation rate of 1.91 kg m^−2^ h^−1^ was calculated from the slope of the curves. Combined with porous microstructure and superwettability, the evaporating abilities of our double-layered evaporator were higher than that of pure water (0.51 kg m^−2^ h^−1^) or single-layered PNFs-PPy (0.79 kg m^−2^ h^−1^) (Fig. S17). In addition, the efficiency was calculated to be 85% at the sun density of 1 kW m^−2^. Such performance ranks into the top-class interfacial solar evaporators that reported so far [69–76] (Fig. 6c). Figure 6d presents the photo of a floating solar evaporator, placed in an upper open container of brine. Driven by the solar-thermal conversion, the generated heat vaporized the underlying brine water immediately, and then condensed into liquid water flowing to the bottom of the transparent chamber. To prove the solar desalination capability of the optimized evaporator, artificial seawater with an average salinity of 3.5 wt% was used. After evaporation–condensation process, the salinity of the distilled water was significantly decreased (Fig. S18), and was even below the drinking water standards (determined by the World Health Organization (WHO)) by approximately two orders of magnitude [77]. Moreover, the concentration of all four primary ions of Na^+^, K^+^, Mg^2+^, and Ca^2+^ that presented in the seawater were reduced significantly (Fig. 6e), and were below the values obtained through reverse osmosis techniques [46, 78]. In particular, the sodium concentration (~ 5 mg L^−1^) is far below the threshold that proposed by WHO guidelines (200–250 mg L^−1^), benefitting the health protection by decreasing sodium intake [79].

Durable evaporation performance is important for long-term practical applications. As shown in Fig. 6f, a linear mass loss of water was recorded when the evaporator was continuously illuminated under 1 sun over 10 h. In this process, the steady-state evaporation rate and solar conversion efficiency were found to exhibit small variation. On the basis of the high-performance of our solar evaporator, outdoor solar desalination was performed to demonstrate its practical applicability (Fig. 6g). The experiment was conducted from 10:00 − 18:00 under natural sunlight with solar intensity ranged from 57 to 1000 W m^−2^, and the air velocity varied between 3.3 and 5.3 m s^−1^. In particular, a maximum evaporation rate of 4.20 kg m^−2^ h^−1^ was achieved at 14:00, the increased evaporation might be attributed to the synergistic effect of sunlight intensity and air velocity (Fig. S19). Even under the non-ideal conditions (low solar irradiation), the evaporator still enabled solar water purification, revealing its potential for real applications.

**Fig. 7:**
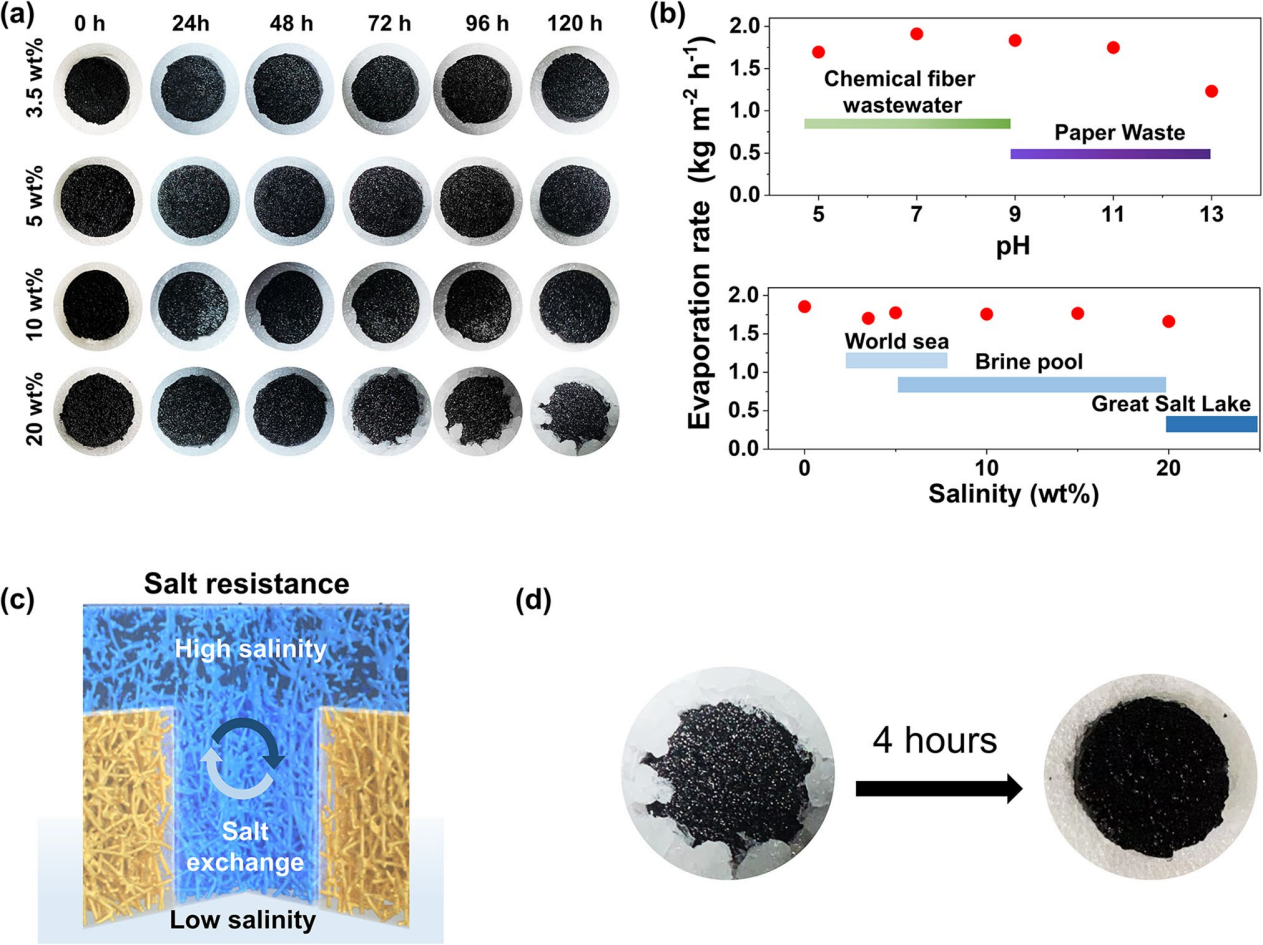
Salt rejection performance. **a** Digital photoes showing salt-resistance of our evaporator with 120 h-continuous operation. **b** Endurance measurement at the extreme pH and high salinity. **c** Working mechanism of salt diffusion in our evaporators for self-cleaning. **d** Digital photos showing salt resistance of our evaporator even a large amount of salt crystals accumulated on the edge of the evaporator

For long-term practical applications, salinity tolerance is another important aspect of solar evaporation [80, 81]. During the continuous working under solar illumination, the salinity on the surface of the evaporator would inevitably increase, the aggregation of salt crystals on the surface of the solar absorber in usually occurred [82]. In contrast to the previous reported systems, a clean top surface without salt aggregation was observed during the 120 h of continuous water desalination (Fig. 7a). Even in high salinity brines (20 wt%), salt crystals were only accumulated on the edge of the evaporator to ensure the continuous solar evaporation. In addition, evaporation rate is independent of salinity (0–20 wt%), and varied little in a broad pH region of 5–13 (Fig. 7b), indicating stable performance under extreme conditions[15]. The durable performance of our evaporators could be ascribed to the robust chemical bonding among polysiloxane and BC nanofibers. Due to the superhydrophilic wettability and porous structure, PNFs could transport salt from the material surface (high salinity) back to underlying water (low salinity) through the water pathways (Fig. 7c) [83]. Such phenomenon could be described as the competition between the salt excretion and diffusion. The salt excretion rate (*J*_excr_, kg m^−2^ h^−1^) and the salt ions diffusion performance (*J*_diff_, kg m^−2^ s^−1^, Fick’s law of diffusion) could be calculated as following equations:

2 $$J_{excr} = \eta \left( {\frac{{q_{0} }}{{h_{lv} }}} \right) \cdot \frac{{C_{b} }}{{\rho_{water} }}$$

3 $$J_{diff} = - D\frac{d\varphi }{{dx}} = \frac{{\varepsilon D\left( {C_{s} - C_{b} } \right)}}{\tau L}$$

*q*_*0*_ is the solar intensity*, h*_lv_ is the liquid–vapor phase change enthalpy, *D* is 1.6 × 10^−9^ m^2^ s^−1^ (NaCl diffusion coefficient in water); *C*_s_ and *C*_b_ are the ions concentrations in PNFs-PPy and bulk solution (salinity = 25 wt%, saturated brine), respectively; *ε* is the porosity of the evaporator (= 99.5%); *τ* is the tortuosity of water path (*τ* = 1.0 for porous material), $L$ is the length of the water path (for our system, *L* = 2.0 cm) [68]. According to the above equations, it is calculated that the value of *J*_diff_ is larger than that of *J*_excr_ in our evaporator system, quantitatively demonstrating the achievement of sufficient salt transfer. In the absence of sunlight, the salt crystals accumulated on the edge of the evaporator could be dissolved by unsaturated water from pores of our evaporator within 4 h (Figs. 7d and S20, Movie S4). From above results, the solar-driven interfacial evaporation integrated by the tunable superwettability aerogels is efficient, low cost, structural robustness, and convenient maintenance.

## Conclusions

In summary, we prepared superhydrophobic and superhydrophilic polysiloxane-strengthened aerogels (VNFs and PNFs) through assembling different groups exposed on the surface of BC nanofibrous framework. The molecular orientation was mediated by the hydrogen bonding between hydrophilic units of siloxane (Si–OH) and the hydroxyl groups (-OH) on the skeleton of BC nanofibers. Further, superhydrophobic VNFs and superhydrophilic PNFs were integrated into a double-layered evaporator, showing high-performance solar-driven interfacial evaporation as follows: (1) with the different surface wettability and superposed architecture, our aerogel evaporator exhibits excellent water evaporation rates of 1.91 and 4.20 kg m^−2^ h^−1^ under laboratory and outdoor solar conditions (1 sun irradiation), which are among the best-performance aerogel evaporators reported so far; (2) by virtue of structural elasticity and interfacial robustness, the aerogel evaporators possess excellent mechanical properties at the 1000th compression cycle, showing long-term durability even under extreme conditions; (3) taking advantages of the superwettable property and porous structure, our evaporator exhibit excellent salt-resistance under continuous operation. Apart from solar water purification, our single component-based strategy would inspire more attention on synthesis of materials with tunable properties for various targeted applications.

### Supplementary Information

Below is the link to the electronic supplementary material.

Supplementary file1 (PDF 2549 kb)

Supplementary file2 (MP4 29546 kb)

Supplementary file3 (MP4 841 kb)

Supplementary file4 (MP4 7510 kb)

Supplementary file5 (MP4 54513 kb)
