# Supplementary material for: Fibrous Aerogels with Tunable Superwettability for High-Performance Solar-Driven Interfacial Evaporation
Source: Nanomicro Lett. 2023 Mar 10;15:64. doi: 10.1007/s40820-023-01034-4 (PMC10006392; doi:10.1007/s40820-023-01034-4)
Supplement: Supplementary file 1 — Supplementary file1 (PDF 2549 kb) [file 40820_2023_1034_MOESM1_ESM.pdf]

Supporting Information for

Fibrous Aerogels with Tunable Superwettability for High-Performance Solar-Driven Interfacial Evaporation

Chengjian Xu[1], Mengyue Gao[1], Xiaoxiao Yu[1], Junyan Zhang[1], Yanhua Cheng[1, *], Meifang Zhu[1, *]

[1]State Key Laboratory for Modification of Chemical Fibers and Polymer Materials, College of Materials Science and Engineering, Donghua University, Shanghai 201620, P. R. China

*Corresponding authors. E-mail: cyh@dhu.edu.cn (Yanhua Cheng); zmf@dhu.edu.cn (Meifang Zhu)

Supplementary Figures and Table

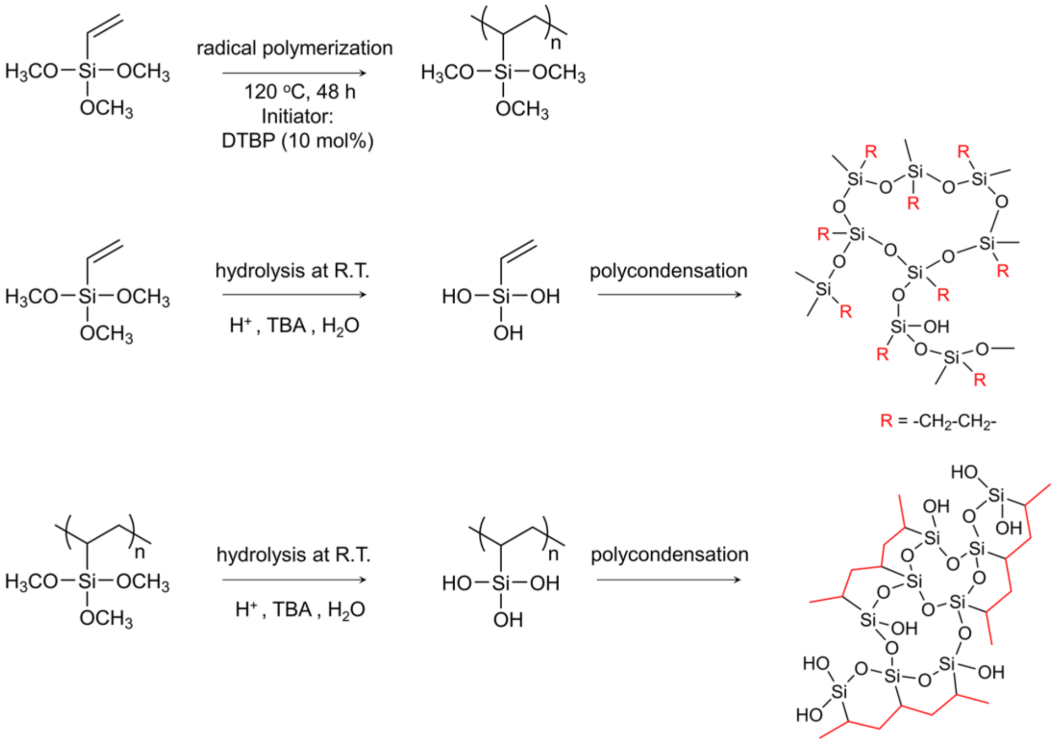

Fig. S1 Synthetic routes of polysiloxane via free-radical polymerization and hydrolytic polycondensation

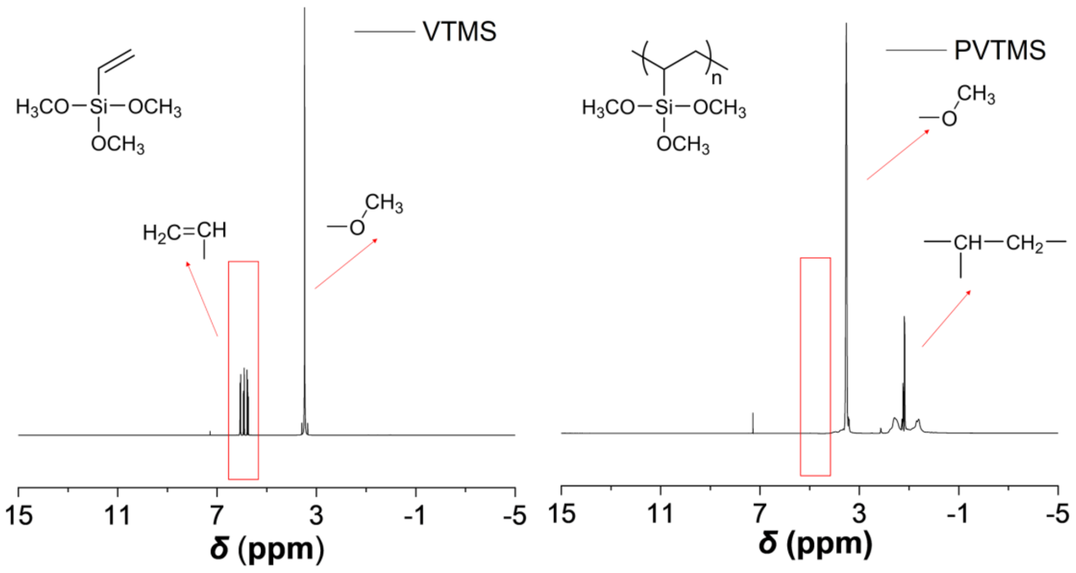

Fig. S2 ^{1}H NMR spectra of (left) monomer and (right) polymer precursors

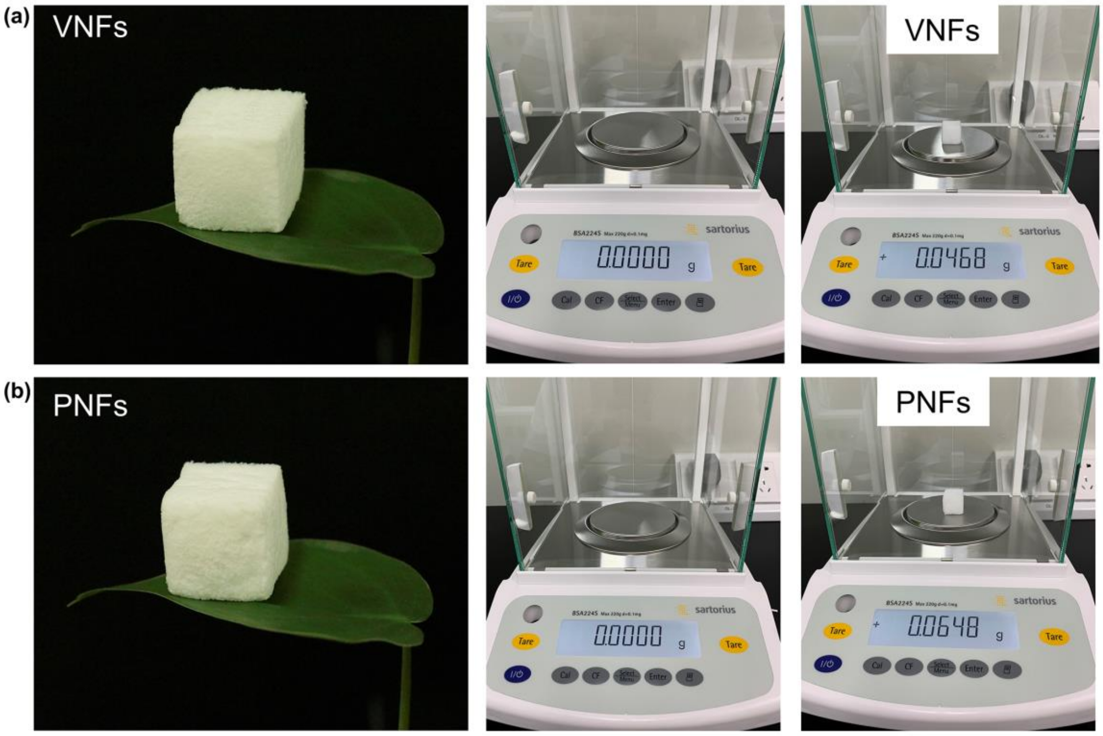

Fig. S3 a-b Photographs of lightweight aerogels standing on leaves and their weight measurement. The densities of VNFs and PNFs are ~6 mg cm^{-3} and ~8 mg cm^{-3}, respectively. $\rho_{VNFs} = m/V = 46.8$ mg/(2 × 2 × 2 cm^3) = 5.85 mg cm^{-3}; $\rho_{PNFs} = m/V = 64.8$ mg/(2 × 2 × 2 cm^3) = 8.1 mg cm^{-3}; top panel: VNFs; bottom panel: PNFs

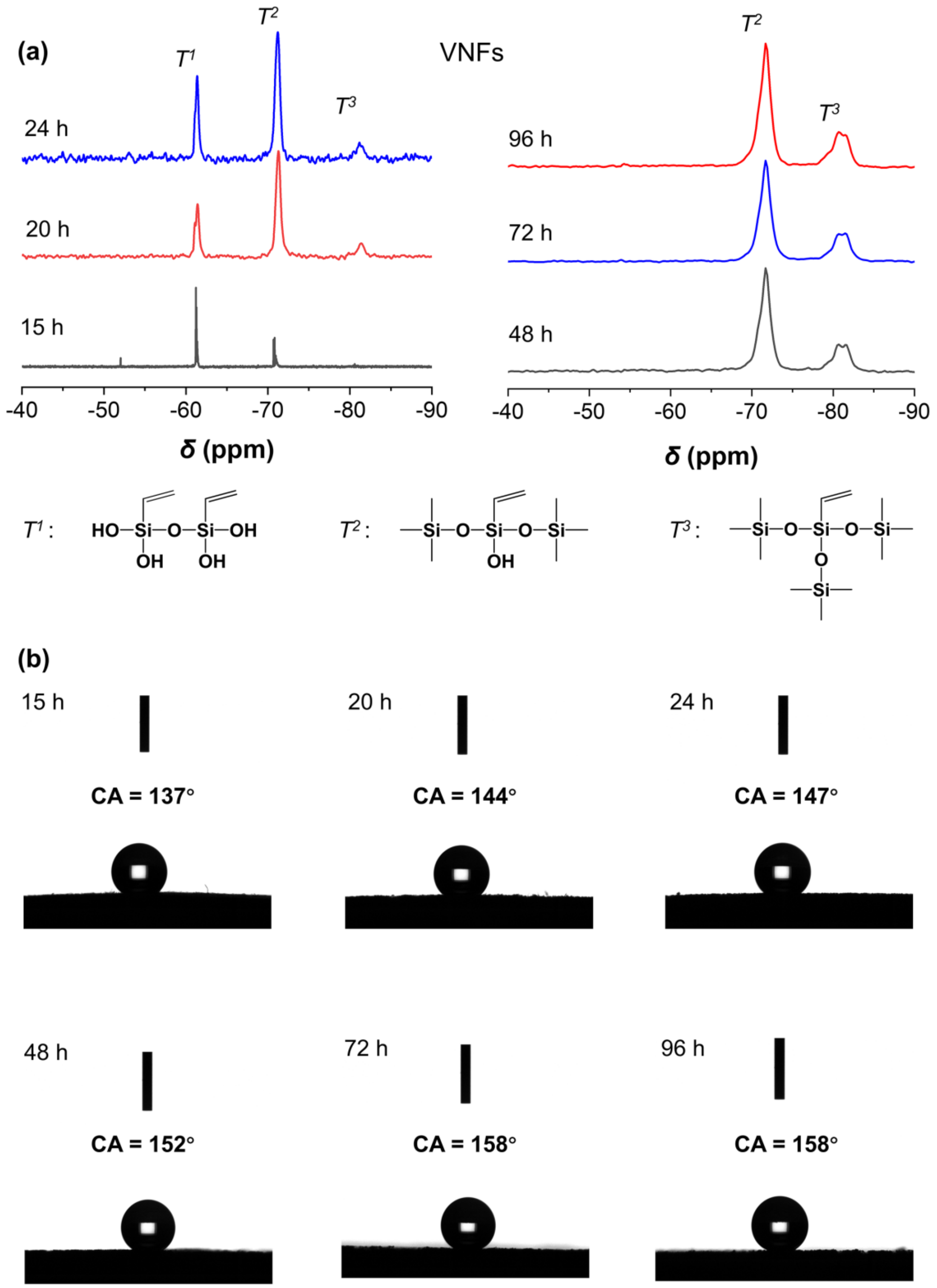

Fig. S4 a-b Solid-state ^{29}Si NMR spectra and water contact angle photos for monitoring polycondensation process of VTMS. Water contact angle results show hydrophobicity of VNFs aerogel was originated at the initial process of materials formation, demonstrating molecular orientation of siloxane was occurred when they contact with BC nanofibers

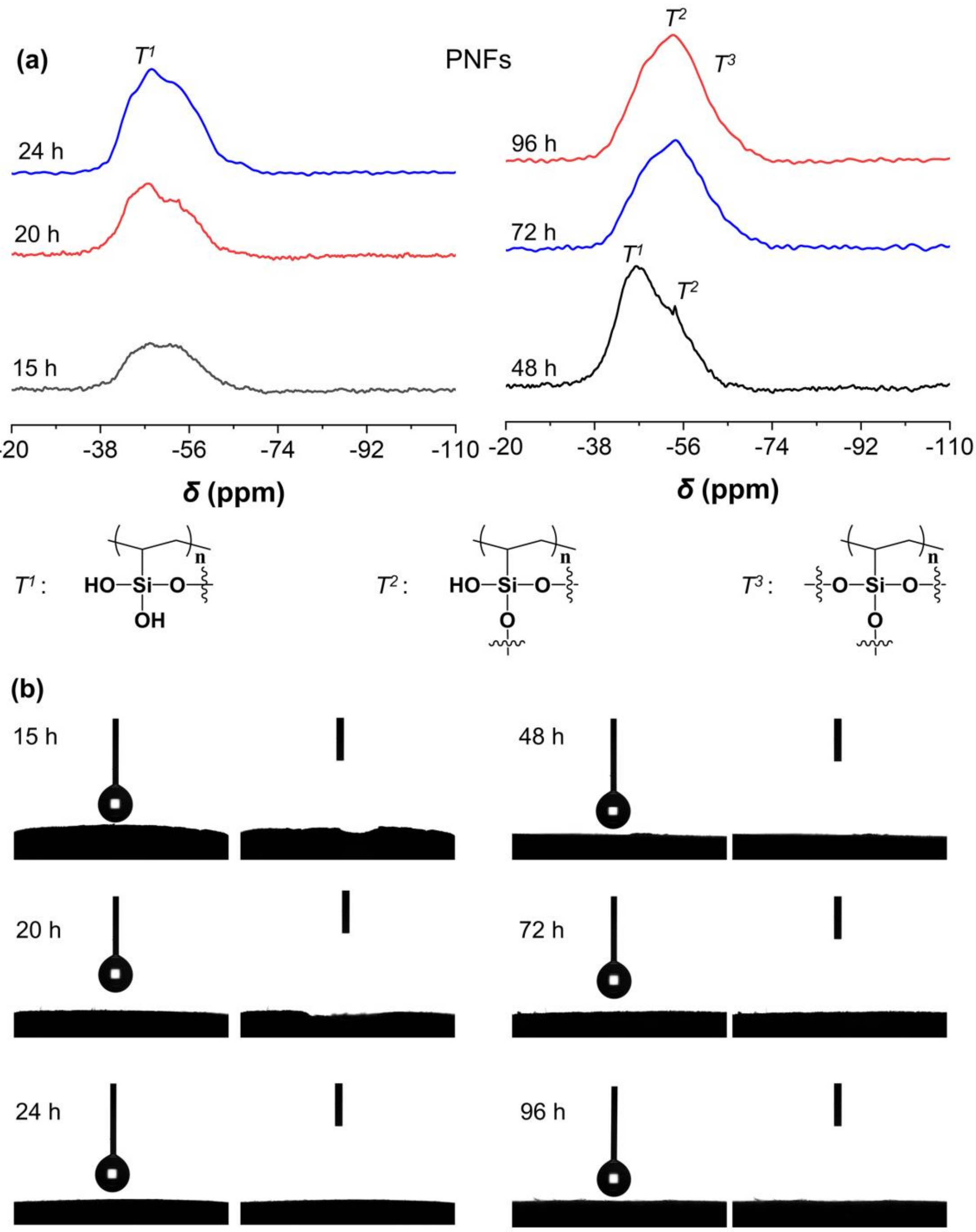

Fig. S5 a-b Solid-state ^{29}Si NMR spectra and water contact angle photos for monitoring the polycondensation process of PVTMS. During the initial process, the structure of the preliminarily-developed aerogel was easily collapsed after water invasion. As polycondensation proceeded, the structure of PNFs became robust and showed negligible deformation after water droplet absorption. The results highlight the capability of polysiloxane that could strengthen the aerogel network

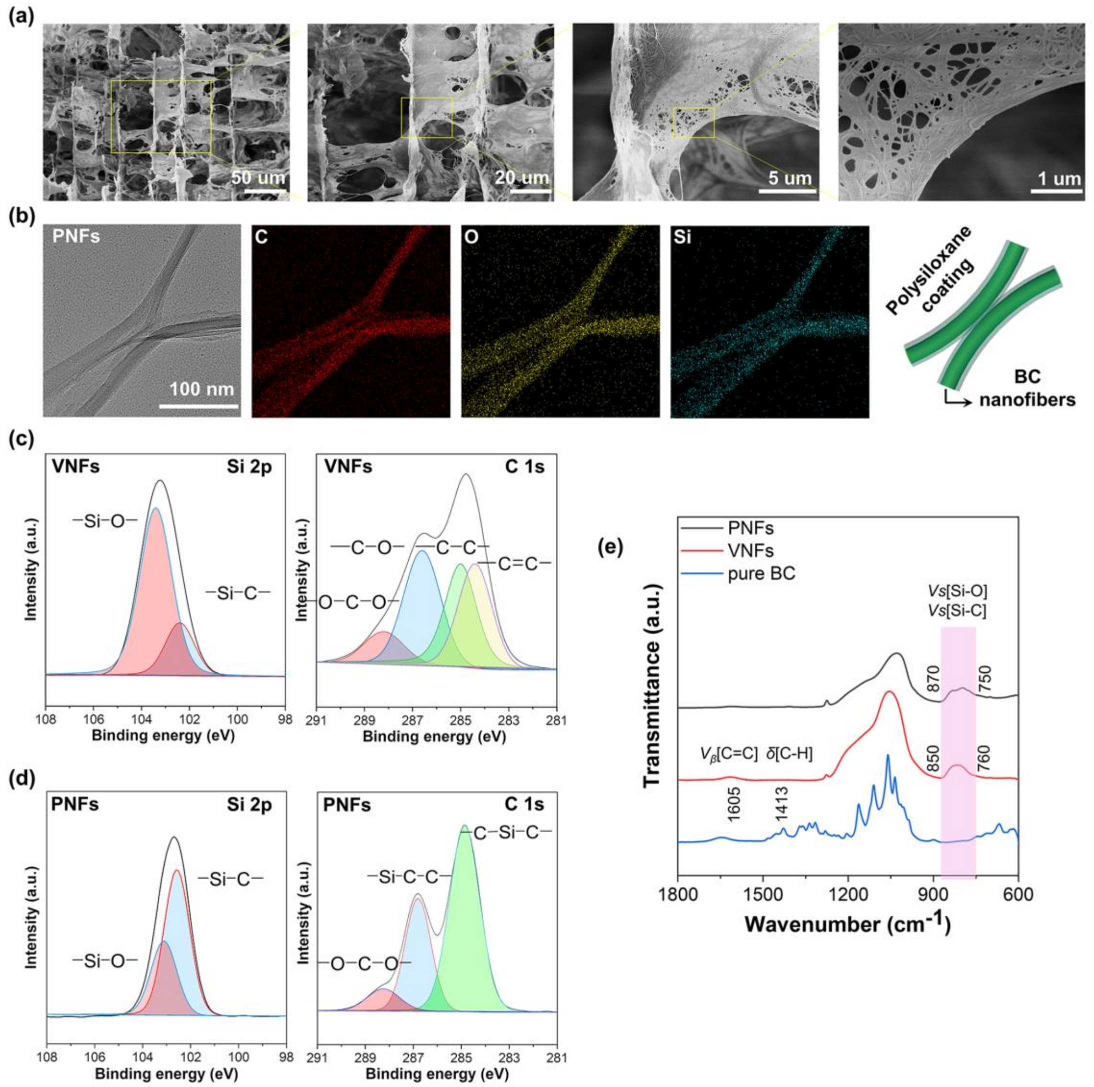

Fig. S6 a Microscopic structure of PNFs observed by SEM at different magnifications. **b** STEM-EDS mapping of nanofibers of PNFs aerogel with elements of C, O, and Si, respectively. **c-d** Si 2p and C 1s XPS spectra of VNFs and PNFs aerogel, respectively. Si 2p: two peaks at 103.4 eV and 102.4 eV of VNFs could be assigned to -Si-O- and -Si-C- groups of PVSQ; and two peaks at 103.1 eV and 102.6 eV of PNFs could be assigned to -Si-O- and -Si-C- groups of PVPSQ. C 1s: peaks at 288.0 eV, 286.5 eV, 285.0 eV and 284.4 eV could be assigned to -O-C-O-, -C-O-, -C-C- and -C=C- groups of VNFs; peaks at 288.2, 286.8, and 284.8 eV could be assigned to -O-C-O-, -Si-C-C- and -C-Si-C- groups of PNFs [S1]. **e** ATR-FTIR spectra of pure BC, VNFs, and PNFs aerogels, respectively. V_S: symmetric stretching vibration; V_β: in-plane stretching vibrations. The characteristic vibrations of PVSQ appear at 1605 cm^{-1} (V_β[C=C]), 1413 cm^{-1} (δ[C-H] in plane), between 850 to 760 cm^{-1} (V_s [Si-C] and V_s [Si-O]), corresponding to the chemical groups of Si-CH=CH_2, C-Si-O and O-Si-O, respectively. The characteristic vibrations of PVPSQ between 870 to 750 cm^{-1} (V_s [Si-C] and V_s [Si-O]), corresponding to the chemical groups of C-Si-O and O-Si-O [S2]

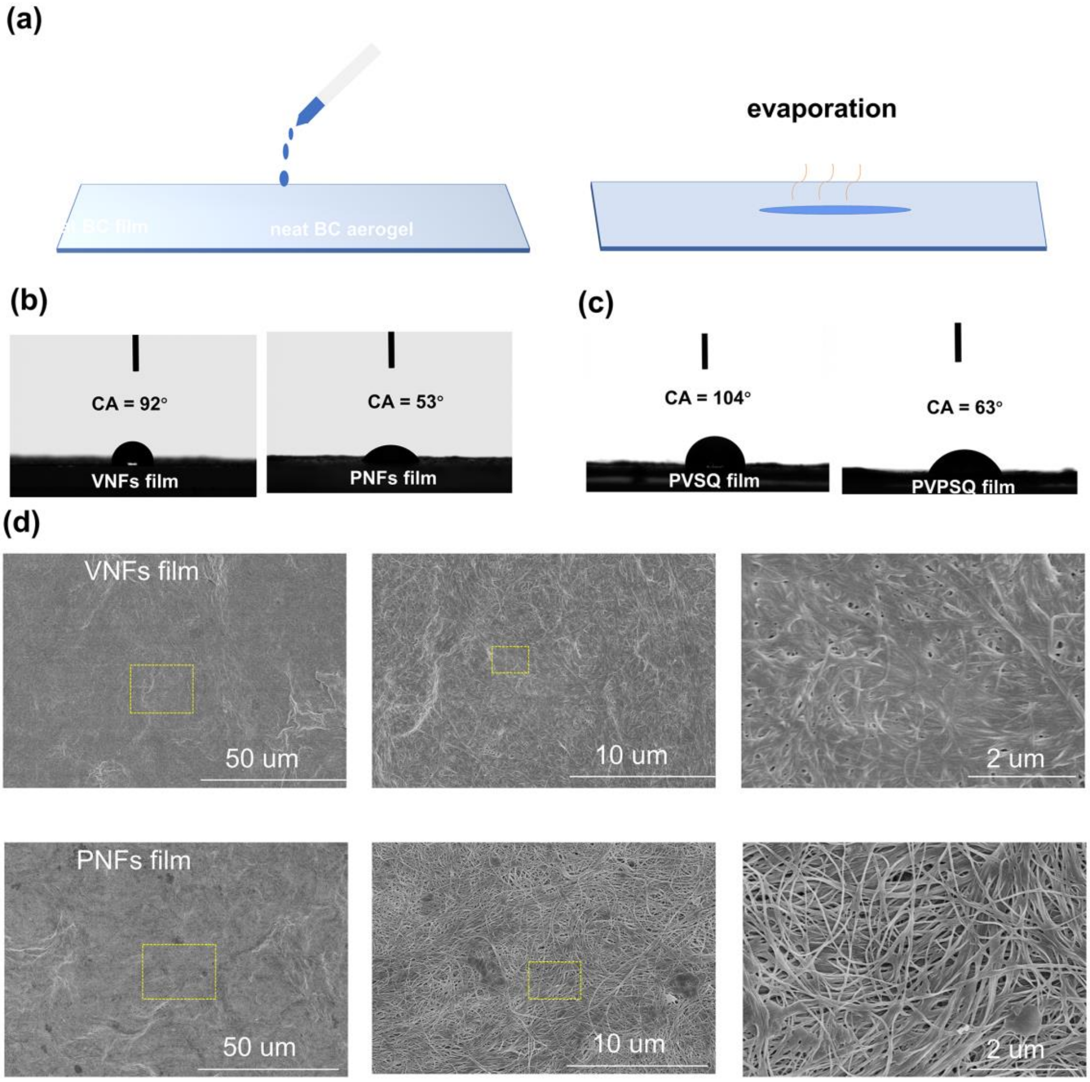

Fig. S7 Controlled experiments for the explanation of superwettability of the aerogels. **a** Schematic illustration of composite films fabricated by dripping the mixture of BC nanofibers and VTMS sol (or PVTMS sol) onto a glass slide, then annealing process at 80 °C for 12 h was applied. Finally, VNFs and PNFs films without porous structure were obtained. **b** Water contact angle of VNFs and PNFs films is 92° and 53°, respectively. The results indicate that surface roughness is important to afford aerogels with superwettable property. **c** Water contact angle of pure PVSQ and PVPSQ film is 104° and 63°, respectively, revealing different molecular orientation of polysiloxane (PVSQ and PVPSQ) for distinct surface wettability. **d** Microscopic structure of VNFs film and PNFs film at different magnifications observed by SEM showing the isotropic fibrous structure that constituted by close-packed nanofibers

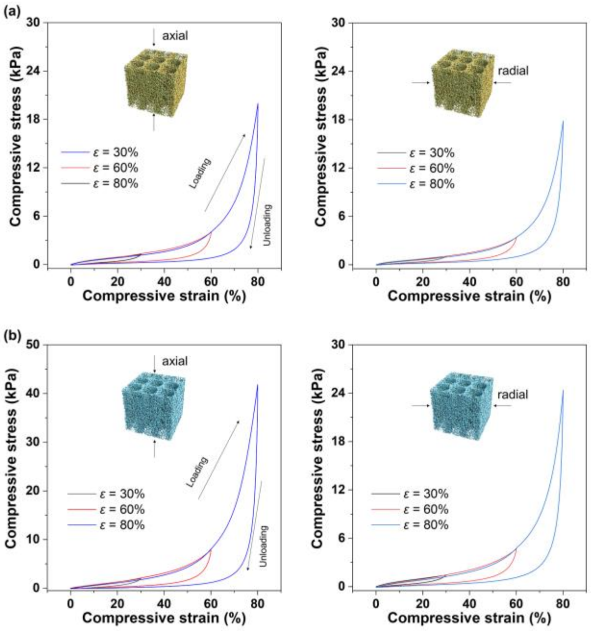

Fig. S8 a-b Compressive stress-strain curves of VNFs and PNFs with incremental strains in (left panel) axial and (right panel) radial directions. From the results, stronger and more stiff mechanical properties were observed in axial direction, showing anisotropic mechanical character of the aerogels. Along the pore alignment, the maximum stress of VNFs and PNFs at the strain of 80% was 20.0 and 41.8 kPa, respectively, indicating that the hybrid aerogels could withstand more than 17,000 times and 27,000 times their own weights.

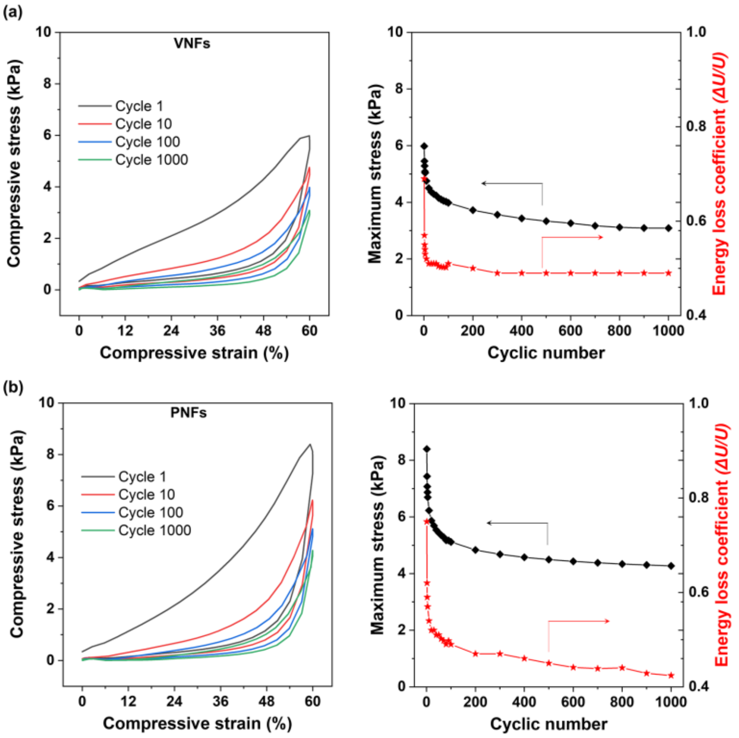

Fig. S9 a-b A 1000-cycle compressive measurement of VNFs and PNFs with ε of 60%, and corresponding maximum stress and energy loss coefficient as a function of compressive cycles. The energy loss coefficient was calculated referring to the literature [S3]

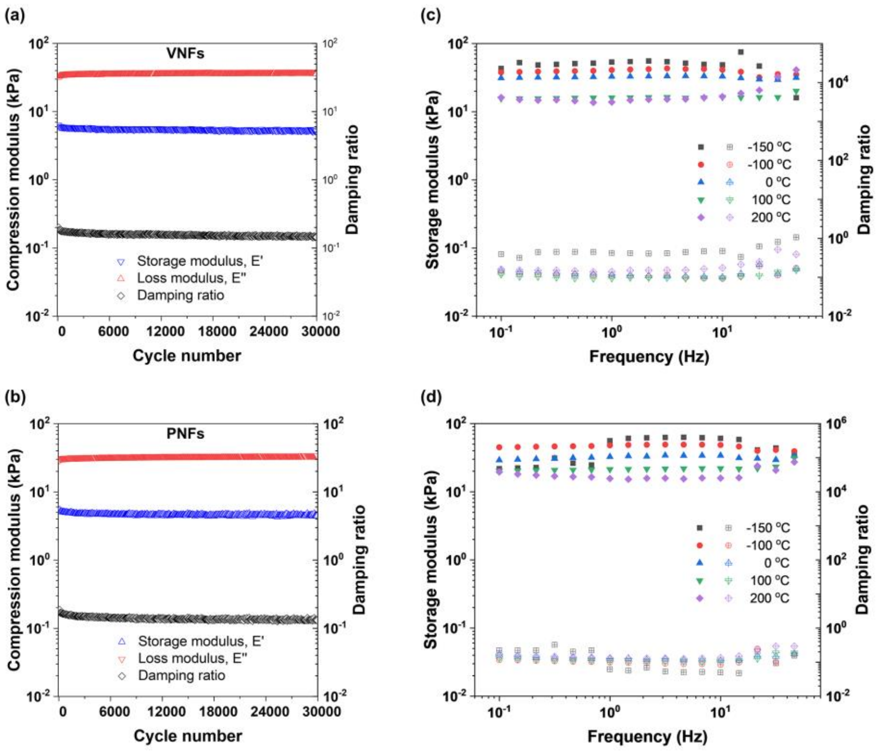

Fig. S10 DMA measurement of VNFs and PNFs (compression mode). **a-b** Fatigue resistance of VNFs and PNFs for 30,000 cycles (± 3% oscillatory strain, 10 Hz, 25 °C, air atmosphere). **c-d** Storage modulus and damping ratio of VNFs and PNFs as a function of frequency (0.1–50 Hz) at temperatures from −150 to 200 °C (air atmosphere)

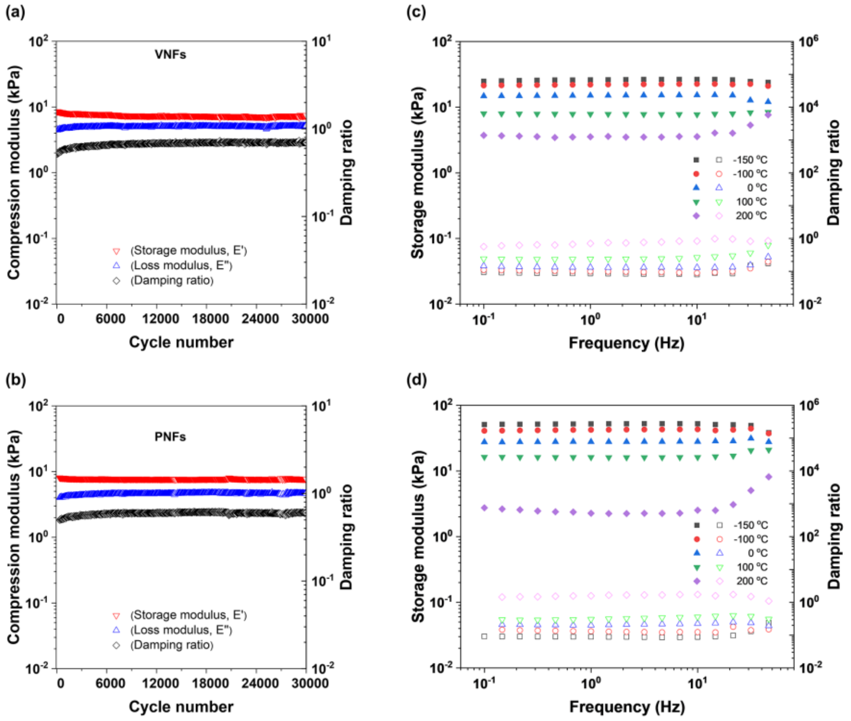

Fig. S11 DMA measurement of VNFs and PNFs (shear mode). **a-b** Fatigue resistance of VNFs and PNFs for 30,000 cycles (± 3% oscillatory strain, 10 Hz, 25 °C, air atmosphere). **c-d** Storage modulus and damping ratio of VNFs and PNFs as a function of frequency (0.1–50 Hz) at temperatures from −150 to 200 °C (air atmosphere)

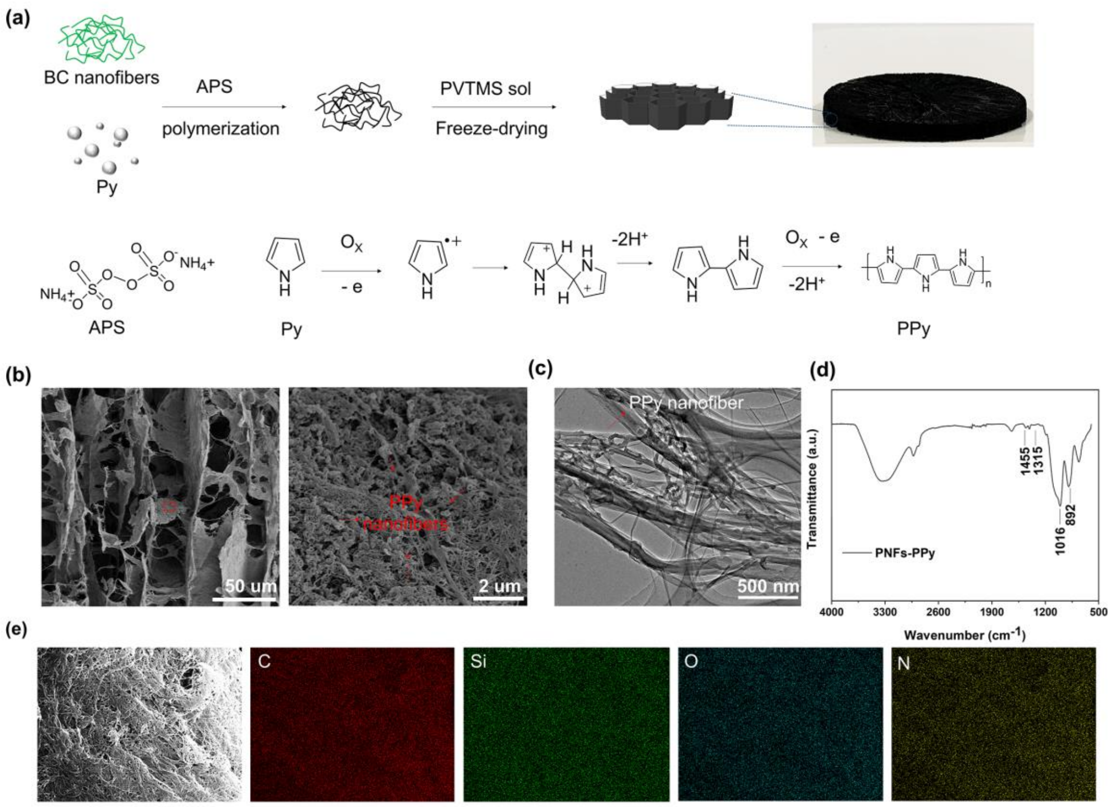

Fig. S12 a Fabrication process of PNFs-PPy. **b** SEM images of PNFs-PPy. PPy nanofibers could be easily found to entangle with BC nanofibrous network. **c** TEM image of PNFs-PPy, confirming the entanglement structure between BC and PPy nanofibers. **d** ATR-FTIR spectra of PNFs-PPy. The symmetric ring-stretching of pyrrole (C-N/C-C stretching) is shown at 1455 cm^{-1}. The peak at 1315 cm^{-1} corresponds to C–N stretching vibration. The band in the region of 1016 and 892 cm^{-1} is attributed to =C–H, N-H in-plane vibration and =C–H out-of-plane vibration, respectively[4, 5]. **e** STEM-EDS mapping of PNFs-PPy nanofibers with elements of C, Si, O, and N, respectively. The elemental mapping of PNF-PPy sample shows the homogeneous distribution of PPy within the aerogel framework

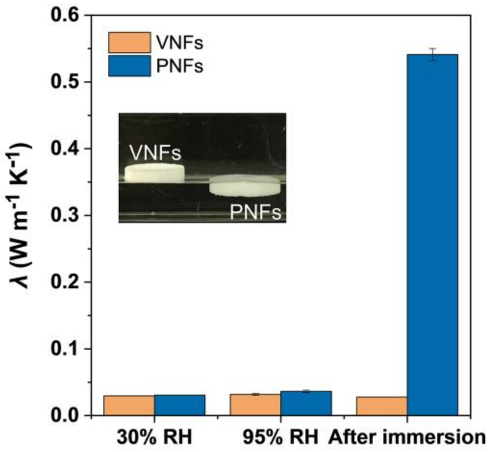

Fig. S13 Thermal conductivities of VNFs and PNFs at 30% and 95% relative humidity (RH), as well as after immersion in water for 8 h. The results indicate that the superhydrophobic VNFs shows stable thermal insulation performance under moisture environment or even in actual application scenarios

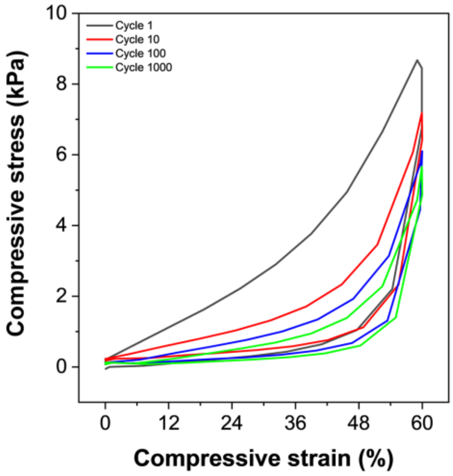

Fig. S14 A 1000-cycle compressive measurement of our double-layered evaporator with 60% strain

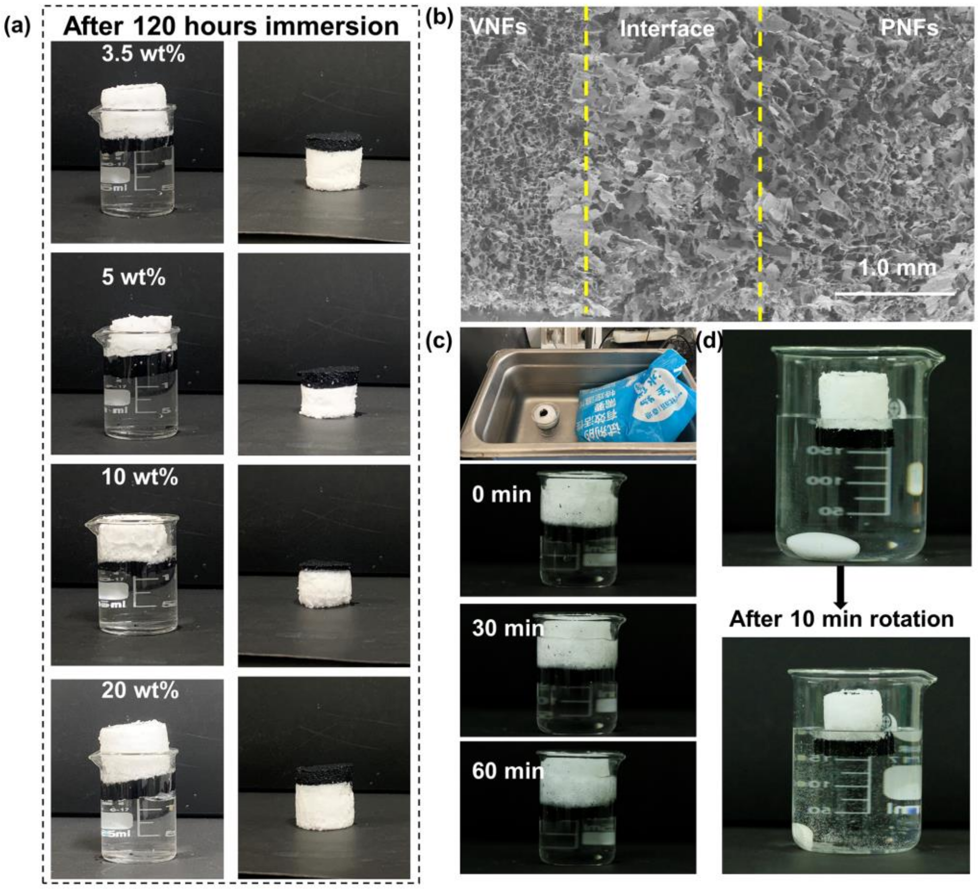

Fig. S15 a Photographs of evaporator showing excellent structural integrity even after 120 h of immersion in four brines at a salinity of 3.5 wt%, 5 wt%, 10 wt%, and 20 wt%, respectively. (Left) Upper part of the evaporators submerged in different brines for 120 h; (right) integrated structure of the double-layered evaporators after immersion. **b** SEM image of the evaporator interface area after 120 h of brine (3.5 wt%) immersion. **c** Photographs of a floating evaporator in simulated seawater (3.5 wt%) at different times under the action of ultrasonication. **d** Photographs of a floating evaporator in simulated seawater (3.5 wt%) before and after 10-min magnetic stirring

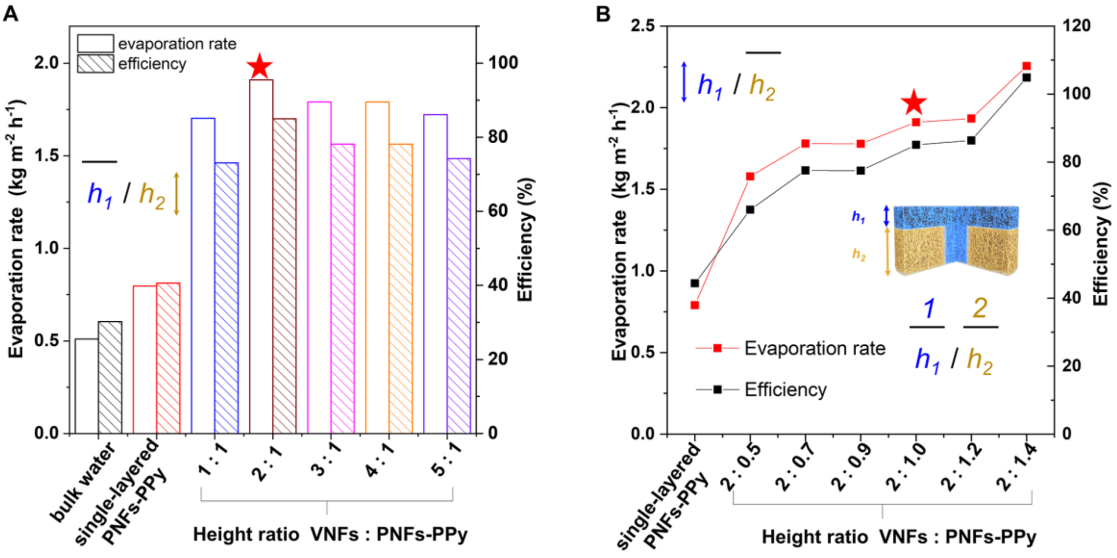

Fig. S16 The heights of top evaporative layer (PNFs-PPy, h_1) and bottom insulation part (VNFs, h_2) were optimized by evaluating evaporation rates and evaporation efficiencies. **a** Water evaporation rates and efficiencies of the evaporators with various architecture. The height of PNFs-PPy (h_1) is fixed to be 1.0 cm, and h_2 is variable. The results revealed that the optimized performance was achieved when h_2 = 2.0 cm. **b** Water evaporation rates and efficiencies of the evaporators with various architecture. Height of VNFs (h_2) was determined to be 2.0 cm, and h_1 is variable. Based on the data in **Fig. S16b**, optimized h_1 and h_2 were determined to be 1.0 and 2.0 cm for evaporator construction. The evaporation efficiencies of above samples are calculated according to Eq. 1 (main text) and the evaporation rate in dark situation was subtracted (0.43 kg m^{-2} h^{-1} for all double-layered evaporators).

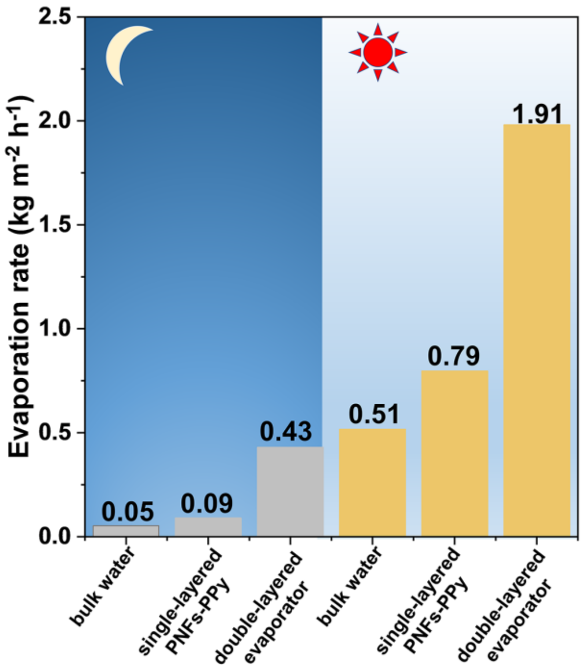

Fig. S17 Water evaporation rates of bulk water, single-layered PNFs-PPy, and the double-layered evaporator (with optimized architecture) in dark and light conditions

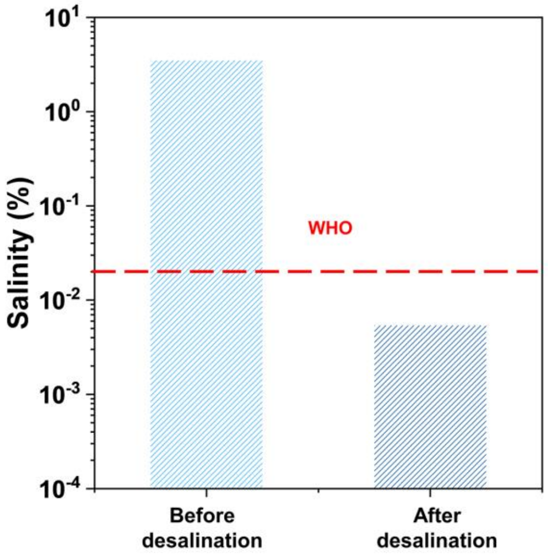

Fig. S18 The salinity of sample before and after desalination

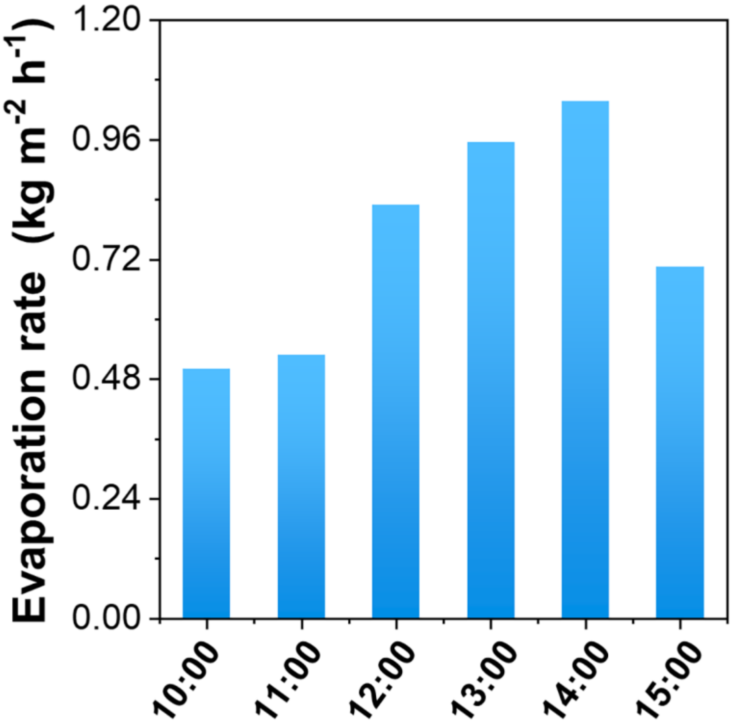

Fig. S19 Water evaporation rates during 6 h of solar desalination at outdoor windless environment. The experiment was conducted in a closed system from 10:00-15:00 under natural sunlight. It is noted that the evaporation rate maximum could reach 1.03 kg m^{-2} h^{-1}, which is much lower than that under windy environment (4.02 kg m^{-2} h^{-1}). On one hand, the air movement allowed the water to evaporate more freely in the open system. On the other hand, open system allowed complete sunlight incidence onto the evaporator surface without energy loss. Both reasons account for higher evaporation rate in an open system under a windy environment.

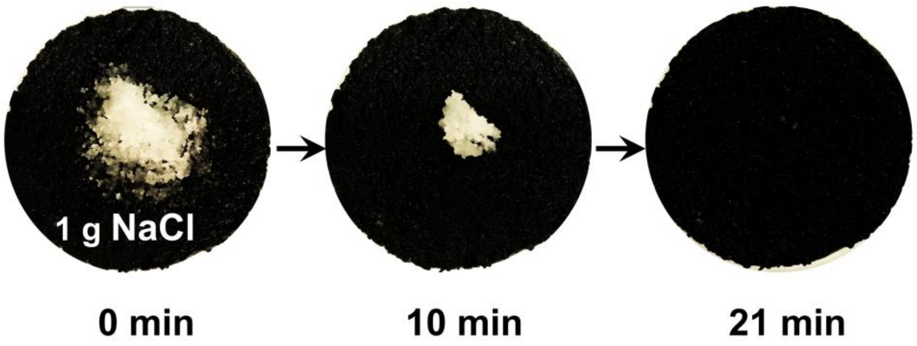

Fig. S20 Digital photos showing the salt diffusion performance of evaporator for 21 min, and the salt crystal was diffused to bulk water through water path

Supplementary Experiment and Discussion

Note S1 Porosity Calculations

The porosity was calculated according to Eq. S1:

$$\boldsymbol{porosity}[\%] = \left(\mathbf{1} - \frac{\boldsymbol{\rho}}{\boldsymbol{\rho_{skeleton}}}\right) \times \mathbf{100\%} \quad \textbf{(S1)}$$

where ρ is the bulk density of aerogel, which can be calculated by the weight and volume of aerogel. ρ_{skeleton} is the density of solid skeleton of aerogel, which can be calculated from Eq. S2:

$$\boldsymbol{\rho_{skeleton}} = \frac{\mathbf{1}}{\frac{\boldsymbol{W_{Si}}}{\boldsymbol{\rho_{Si}}} + \frac{\boldsymbol{W_{BC}}}{\boldsymbol{\rho_{BC}}}} = \frac{\mathbf{1}}{\frac{\boldsymbol{W_{Si}}}{\boldsymbol{\rho_{Si}}} + \frac{\mathbf{1}-\boldsymbol{W_{Si}}}{\boldsymbol{\rho_{BC}}}} \quad \textbf{(S2)}$$

W_{si} is the mass fraction of polysiloxane in the aerogel, which was calculated from Eq. S3:

$$\boldsymbol{W_{Si}}[\%] = \left(\mathbf{1} - \frac{\boldsymbol{m_{BC}}}{\boldsymbol{m}}\right) \times \mathbf{100\%} \quad \textbf{(S3)}$$

where m_{BC} (0.0236 g) and m are the weight of the BC nanofibers (pure BC aerogel) and the entire aerogels (VNFs or PNFs), respectively. ρ_{BC} = 1.50 g cm^{-3}, ρ_{VNFsSi} = 1.18 g cm^{-3}, and ρ_{PNFsSi} = 1.50 g cm^{-3}, which were calculated referring to the literature [S1, S6, S7]. As a result, the porosity of VNFs and PNFs was calculated to be 99.6% and 99.5%, respectively.

Note S2 Simulation Details and Methodology

Classic molecular dynamics simulations were carried out to investigate the molecular orientation of polysiloxane on the surface of BC nanofibers at the atomic level. Two kinds of macromolecules named PVPSQ and PVSQ were studied in this project, which contained saturated carbon chains and vinyl groups, respectively. The molecular structures of PVPSQ, PVSQ, and cellulose are shown in the Figure 3 (main text). Two simulations were performed, in which thirty-five PVSQ molecules (or thirty-five PVPSQ molecules) and thirty-five cellulose molecules were packed in the cubic simulation box with the software of PACKMOL, respectively [S8]. The detailed information about the simulation boxes was listed in Table S1 and referred to the previous study [S9]. For each simulation, energy minimization was firstly employed to relax the simulation box. Next, an isothermal-isobaric (NPT) ensemble with a 1.0 fs time step was taken to optimize the simulation box, where the temperature and pressure

were set to be 298.15 K and 1.0 atm, respectively. The Nose-Hoover thermostat and Parrinello-Rahman barostat were used to stabilize the temperature and pressure of the simulation box. Following the NPT simulation, a canonical (NVT) ensemble with a 30.0 ns time step was performed, in which the prior 20.0 ns was used to optimize the simulation box and the later 10.0 ns was used to collect the trajectory coordinates of molecules. All simulations were carried out using the GROMACS 2019.5 package [S10].

Table S1 The initial configuration setup for each simulation system

	Molecule	Number	Pressure (atm)	Temperature (K)	Initial box size (nm)
System 1	PVPSQ	35	1.0	298.15	8.0
	cellulose	35			
System 2	PVSQ	35	1.0	298.15	8.0
	cellulose	35			

Note S3 Water Transportation Rate Calculation

According to Movie S2, the transport distance of 2.0 cm could be reached (diameter = 1.0 cm) within 2.0 s. Therefore, the volume of water transportation through the water channel could be calculated with Eq. S4:

$$\boldsymbol{V = \pi r^2 \times h \times \frac{3600}{2}} \qquad \textbf{(S4)}$$

The rate of water transportation in PNFs channel was 2827 cm^3 h^{-1}.

On the other hand, the evaporation rate was 1.91 kg m^{-2} h^{-1}, and the diameter of evaporation surface was 5.50 cm, the evaporation rate of the individual evaporator reached 859 cm^3 h^{-1}. Therefore, water supply of the system is sufficient for evaporation, which is important for highly efficient solar evaporator.

Note S4 Energy Loss Analysis

Energy loss of evaporator was analyzed referring to the literature [S11, S12].

(1) Radiation loss was calculated by Stefan-Boltzmann Eq. S5:

$$\boldsymbol{\phi = \varepsilon A \sigma (T_1^4 - T_2^4)} \qquad \textbf{(S5)}$$

Φ is the heat flux, ε is the emissive rate (using value of 1 as the emissivity of evaporator), A is the evaporation aera (23.76 cm^2), σ is the Stefan-Boltzmann constant (σ = 5.67x10^{-8} W m^{-2} K^{-4}), T_1 is the temperature of the absorber (308.5 K for double-layered evaporator, 315.0 K for single-layered PNFs-PPy), and T_2 is the ambient temperature of evaporator (= 306.5 K).

According to above data, the radiation loss of double-layered evaporator was calculated to ~3.0%, and ~13.7% for single-layered PNFs-PPy.

(2) Conduction loss was calculated by Eq. S6:

$$\boldsymbol{Q = Cm\Delta T} \qquad \textbf{(S6)}$$

Q is the heat energy, C is the specific heat capacity of pure water (= 4.2 kJ °C^{-1} kg^{-1}), m is the weight of bulk water, ΔT is the temperature difference between the bulk water before and after experiment. In our experiment, the weight of bulk water was 60.0 g, the temperature difference of double-layered evaporator reached 0.8 °C, while 6.0 °C

for single-layered PNFs-PPy. According to above data, the conduction heat loss of the double-layered evaporator was calculated to be ~2.3%, and ~17.6% for single-layered PNFs-PPy.

(3) Convection loss was calculated by Eq. S7:

$$P_{conv} = hA_{surface}\Delta T \qquad \text{(S7)}$$

h is the convection heat transfer coefficient (~5 W m^{-2} K^{-1}), $A_{surface}$ is the surface aera of evaporator, ΔT is the temperature difference between evaporator surface and ambient environment. According to above data, the convection loss of double-layered evaporator was calculated to be ~2.3%, and ~10% for the single-layered PNFs-PPy.

(4) Reflection loss:

The absorption of evaporator according to UV-vis-NIR diffuse reflectance spectroscopy (Figure 4c in main text) is 95%; thus, the refection loss of double-layered evaporator and single-layered PNFs-PPy is ~5%.

Note S5 Water Evaporation Enthalpy Calculation

The water vaporization enthalpy of water-filled PNFs-PPy aerogel was determined by the equivalent evaporation enthalpy measurement. The experimental details were referred to the literature and calculated using Eq. S8 [S13]:

$$U_{in} = \Delta H_{vap}m_0 = \Delta H_{equ}m_g \qquad \text{(S8)}$$

where ΔH_{vap} is the evaporation enthalpy, and m_0 is the mass change of bulk water; ΔH_{equ} and m_g are the equivalent evaporation enthalpy and mass change of water-filled PNFs-PPy aerogel, respectively. According to the above equation, the water evaporation enthalpy of water in water-filled PNFs aerogel was calculated to be 2068 J g^{-1}.

Note S6 Optimized Geometry

The height of VNFs and PNFs-PPy was determined by gravity, heat location performance, and salt rejection performance referring to the literature [S11, S14].

The single-layered PNFs-PPy evaporator was immersed in water throughout the evaporation process, resulting in heat loss to bulk water. As a result, VNFs was introduced to support PNFs-PPy. The force required to support PNFs-PPy was derived from the water excluded by VNFs, and was calculated by Eq. S9:

$$F_b = G_{ex} = \rho_w \cdot g \cdot V_{ex} \qquad \text{(S9)}$$

F_b represents the buoyancy provided by VNFs, G_{ex} is gravity of the water (V_{ex}: volume of water) excluded by the double-layered evaporator, and ρ_w corresponds to density of water. The critical condition of the suitable design is that F_b should be equal to G_{ex}. Therefore, the following Eqs. S10-S12 are derived:

$$\rho_w \cdot g \cdot V_{ex} = m_{total} \cdot g \qquad \text{(S10)}$$

$$V_{ex} = \pi \cdot r_1^2 \cdot h_{ex} \qquad \text{(S11)}$$

$$m_{total} = \pi \cdot r_1^2 \cdot h_1 \cdot \rho_1 + \pi \cdot r_2^2 \cdot h_{ex} \cdot \rho_1 + \pi \cdot (r_1^2 - r_2^2) \cdot h_{ex} \cdot \rho_2 \quad \text{(S12)}$$

In this study, ρ_1 = 1.00 g cm^{-3}, ρ_2 = 6.00 mg cm^{-3}. h_{ex} can be simplified using Eq. S13:

$$h_{ex} = \frac{r_1^2 \cdot h_1}{(1-\rho_2)(r_1^2 - r_2^2)} \quad \text{(S13)}$$

In our system, r_1 = 0.50 cm, r_2 = 2.75 cm, h_1 = 1.00 cm. h_{ex} was calculated to be 1.04 cm. Therefore, as long as h_2 > 1.04 cm, solar absorber is able to isolate the bulk water from PNFs-PPy layer.

From the point of heat location, the thickness of insulator is important in reducing heat conduction from top to bulk water and determining the height of water path. Heat transferred by the conduction process could be expressed by the Eqs. S14 and S15:

$$dQ = -\lambda dS \frac{\partial t}{\partial n} \quad \text{(S14)}$$

$$Q = \lambda A \frac{\Delta t}{\Delta x} \quad \text{(S15)}$$

Q is the heat transferred through the system, λ is the thermal conductivity of VNFs, Δt is the temperature difference between top and bottom of evaporator, A is the area of the surface, and Δx is the thickness of VNFs.

In this system, we define that the heat conduction loss through the insulator could be less than 5%, and the temperature difference between top and bottom is 10 °C, that is

$$\frac{Q}{A} \leq 5\%\ q_0\ ,\ \Delta t = 10\ ℃$$

Therefore, Δx should be larger than 0.60 cm, which is also the length of water path.

$$\Delta x \geq 0.60\ cm$$

For salt rejection performance, the length of water path determines the diffusion performance of salt ions (J_{diff}, kg m^{-2} s^{-1}) according to Eq. S16 (Fick's law of diffusion) [S14] :

$$J_{diff} = -D\frac{d\varphi}{dx} = \frac{\varepsilon D(C_s - C_b)}{\tau L} \quad \text{(S16)}$$

D is 1.6 × 10^{-9} m^2 s^{-1} (NaCl diffusion coefficient in water); C_s and C_b are the ions concentrations in PNFs-PPy and bulk solution (salinity = 25 wt%, saturated brine), respectively; ε is the porosity of the evaporator (= 99.48%); τ is the tortuosity of water path (= 1), L is the length of the water path. With a salt excretion rate (J_{excr}, kg m^{-2} h^{-1}) on the evaporator under 1 sun illumination (1 kW m^{-2}), the solar vapor generation energy conversion efficiency could be calculated by Eq. S17:

$$\eta = \frac{m \cdot h_{lv}}{q_0} \quad \text{(S17)}$$

m is the mass flux during evaporation, h_{lv} is the liquid-vapor phase change enthalpy, J_{excr} could be calculated by Eq. S18:

$$J_{excr} = \eta(\frac{q_0}{h_{lv}}) \cdot \frac{C_b}{\rho_{water}} \quad \textbf{(S18)}$$

In order to avoid salt accumulation, J_{diff} should be larger than J_{excr}, and the final length of water path could be calculated with Eq. S19:

$$L \le \frac{h_{lv} \cdot \rho_{water} \cdot \varepsilon \cdot D \cdot (C_s - C_b)}{\eta q_0 \tau C_b} \quad \textbf{(S19)}$$

As a result, the length of water channel (L) was calculated to be less than 2.35 cm.

Combining the optimized length by calculation of gravity, heat conduction, and salt rejection, the length of water path (h_2) should be in the range of 1.04–2.35 cm.

Supplementary References

[S1] L. Wang, J. Feng, Y. Jiang, S. Zhang, L. Li et al., Facile fabrication of hydrophobic polyvinylpolysilsesquioxane aerogels with improved optical properties. J. Sol-Gel Sci. Technol. **94**, 88-97 (2020). https://doi.org/https://doi.org/10.1007/s10971-019-05148-3

[S2] R. Al-Oweini, H. El-Rassy, Synthesis and characterization by FTIR spectroscopy of silica aerogels prepared using several $Si(OR)_4$ and $R''Si(OR')_3$ precursors. J. Mol. Struct. **919**, 140-145 (2009). https://doi.org/https://doi.org/10.1016/j.molstruc.2008.08.025

[S3] F. Wang, L. Dou, J. Dai, Y. Li, L. Huang et al., In situ synthesis of biomimetic silica nanofibrous aerogels with temperature-invariant superelasticity over one million compressions. Angew. Chem. Int. Ed. **59**, 8285-8292 (2020). https://doi.org/https://doi.org/10.1002/anie.202001679

[S4] Z. Ahmad, M.A. Choudhary, A. Mehmood, R. Wakeel, T. Akhtar et al., Synthesis of polypyrrole nano/microspheres using cobalt(III) as an oxidizing agent and its ammonia sensing behavior. Macromol. Res. **24**, 596-601 (2016). https://doi.org/10.1007/s13233-016-4081-x

[S5] Y. Cai, J. Shen, C. Yang, Y. Wan, H. Tang et al., Mixed-dimensional MXene-hydrogel heterostructures for electronic skin sensors with ultrabroad working range. Sci. Adv. **6**, eabb5367 (2020). https://doi.org/10.1126/sciadv.abb5367

[S6] T. Shimizu, K. Kanamori, A. Maeno, H. Kaji, C. M. Doherty et al., Transparent, highly insulating polyethyl- and polyvinylsilsesquioxane aerogels: mechanical improvements by vulcanization for ambient pressure drying. Chem. Mater. **28**, 6860-6868 (2016). https://doi.org/10.1021/acs.chemmater.6b01936

[S7] G. Zu, K. Kanamori, A. Maeno, H. Kaji, K. Nakanishi. Superflexible Multifunctional Polyvinylpolydimethylsiloxane-Based Aerogels as Efficient Absorbents, Thermal Superinsulators, and Strain Sensors. Angew. Chem. Int. Ed. **57**, 9722-9727 (2018). https://doi.org/10.1002/anie.201804559

[S8] L. Martínez, R. Andrade, E. G. Birgin, J. M. Martínez. PACKMOL: A package for building initial configurations for molecular dynamics simulations. J. Comput. Chem. **30**, 2157-2164 (2009). https://doi.org/10.1002/jcc.21224

[S9] C. Luo, Y. Li, W. Sun, P. Xiao, S. Liu, D. Wang, C. Zheng. Revisiting the corrosion mechanism of LiFSI based electrolytes in lithium metal batteries. Electrochim. Acta **419**, 140353 (2022). https://doi.org/10.1016/j.electacta.2022.140353

[S10] S. Páll, M. J. Abraham, C. Kutzner, B. Hess, E. Lindahl. Tackling Exascale Software Challenges in Molecular Dynamics Simulations with GROMACS. In *Solving Software Challenges for Exascale*, Cham, 2015, Markidis, S., Laure, E., Eds.; Springer International Publishing: pp 3-27.

[S11] N. Li, L. Qiao, J. He, S. Wang, L. Yu, P. Murto, X. Li, X. Xu. Solar-Driven Interfacial Evaporation and Self-Powered Water Wave Detection Based on an All-Cellulose Monolithic Design. Adv. Funct. Mater. **31**, 2008681 (2021). https://doi.org/10.1002/adfm.202008681

[S12] Z. Liu, H. Song, D. Ji, C. Li, A. Cheney, Y. Liu, N. Zhang, X. Zeng, B. Chen, J. Gao, Y. Li, X. Liu, D. Aga, S. Jiang, Z. Yu, Q. Gan. Extremely Cost-Effective and Efficient Solar Vapor Generation under Nonconcentrated Illumination Using Thermally Isolated Black Paper. Global Challenges **1**, 1600003 (2017). https://doi.org/10.1002/gch2.201600003

[S13] Z. Yu, P. Wu, Biomimetic MXene-polyvinyl alcohol composite hydrogel with vertically aligned channels for highly efficient solar steam generation. Adv. Mater. Technol. **5**, 00065 (2020). https://doi.org/10.1002/admt.202000065

[S14] L. Zang, C. Finnerty, S. Zheng, K. Conway, L. Sun et al., Interfacial solar vapor generation for desalination and brine treatment: Evaluating current strategies of solving scaling. Water Res. **198**, 117135 (2021). https://doi.org/10.1016/j.watres.2021.117135
